# Emerging Mechanisms Underpinning Neurophysiological Impairments in *C9ORF72* Repeat Expansion-Mediated Amyotrophic Lateral Sclerosis/Frontotemporal Dementia

**DOI:** 10.3389/fncel.2021.784833

**Published:** 2021-12-15

**Authors:** Iris-Stefania Pasniceanu, Manpreet Singh Atwal, Cleide Dos Santos Souza, Laura Ferraiuolo, Matthew R. Livesey

**Affiliations:** Sheffield Institute for Translational Neuroscience, University of Sheffield, Sheffield, United Kingdom

**Keywords:** *C9ORF72*, neuron, synaptic, excitability, glutamate, physiology, ALS (amyotrophic lateral sclerosis), FTD (frontotemporal dementia)

## Abstract

Amyotrophic lateral sclerosis (ALS) and frontotemporal dementia (FTD) are characterized by degeneration of upper and lower motor neurons and neurons of the prefrontal cortex. The emergence of the *C9ORF72* hexanucleotide repeat expansion mutation as the leading genetic cause of ALS and FTD has led to a progressive understanding of the multiple cellular pathways leading to neuronal degeneration. Disturbances in neuronal function represent a major subset of these mechanisms and because such functional perturbations precede degeneration, it is likely that impaired neuronal function in ALS/FTD plays an active role in pathogenesis. This is supported by the fact that ALS/FTD patients consistently present with neurophysiological impairments prior to any apparent degeneration. In this review we summarize how the discovery of the *C9ORF72* repeat expansion mutation has contributed to the current understanding of neuronal dysfunction in ALS/FTD. Here, we discuss the impact of the repeat expansion on neuronal function in relation to intrinsic excitability, synaptic, network and ion channel properties, highlighting evidence of conserved and divergent pathophysiological impacts between cortical and motor neurons and the influence of non-neuronal cells. We further highlight the emerging association between these dysfunctional properties with molecular mechanisms of the *C9ORF72* mutation that appear to include roles for both, haploinsufficiency of the C9ORF72 protein and aberrantly generated dipeptide repeat protein species. Finally, we suggest that relating key pathological observations in *C9ORF72* repeat expansion ALS/FTD patients to the mechanistic impact of the *C9ORF72* repeat expansion on neuronal function will lead to an improved understanding of how neurophysiological dysfunction impacts upon pathogenesis.

## Introduction

The underlying genetic and pathological causes of amyotrophic lateral sclerosis (ALS) and frontotemporal dementia (FTD) overlap extensively placing them on an ALS-FTD spectrum (Kato et al., 1993; Talbot et al., 1995; Lomen-Hoerth et al., 2002). Clinical observations of ALS-FTD patients reinforce linked pathogenicity where almost half of ALS patients develop FTD-related cognitive disturbances and up to 30% of FTD patients exhibit motor impairment (Christidi et al., 2018). The GGGGCC (G_4_C_2_) hexanucleotide repeat expansion mutation is found within intron 1 of the *C9ORF72* gene (*C9ORF72* repeat expansion, *C9ORF72^RE^*), is causal to both ALS and FTD and is the most common pathogenic mutation within the ALS-FTD spectrum. Degeneration is classically prominent within the pre-frontal cortex in FTD and the motor cortex, upper motor neurons (layer V cortical projection neurons) and lower motor neurons in ALS. Understanding how this mutation mechanistically leads to neuronal injury and degeneration is of key importance.

Healthy individuals typically present with 2–30 G_4_C_2_ repeats whereas ALS-FTD patients living with the repeat expansion typically have hundreds to thousands of repeats, with 65 repeats argued as the pathological repeat-length threshold (DeJesus-Hernandez et al., 2011; Renton et al., 2011). Furthermore, they share TDP-43 pathology that manifests in approximately 98% of ALS patients and 45% of FTD patients (Arai et al., 2006; Neumann et al., 2006). The repeat expansion drives pathogenicity through at least one of two potential broad mechanisms; haploinsufficiency of C9ORF72 protein expression and toxic gain-of-function of the repeat expansion (Gendron et al., 2014; Mizielinska and Isaacs, 2014). The latter can be further subdivided into transcribed repeat expansion sense and antisense RNA foci and aberrant non-ATG (RAN) translation leading to the generation of five potential dipeptide-repeat proteins (DPRs): poly-GA, -GP, -GR, -PA, and -PR (Donnelly et al., 2013; Gendron et al., 2013; Mori et al., 2013). Attention is now focusing on how these mechanisms drive the cellular disturbances observed in ALS-FTD, where emerging research places emphasis on both exclusive and synergistic mechanisms involving haploinsufficiency and aspects of toxic-gain-of-function. Further complexity to our understanding is contributed by the fact we have an incomplete appreciation of the physiological role of the C9ORF72 protein (Smeyers et al., 2021). Importantly, several rodent models initially generated to study *C9ORF72*^RE^ mechanisms do not always recapitulate motor dysfunction (Balendra and Isaacs, 2018), though more recent studies now describe motor deficits in a *C9ORF72* haploinsufficiency model (Shao et al., 2019) and that motor deficits are exacerbated in a background of both haploinsufficiency and the repeat expansion (Zhu et al., 2020). Nonetheless, it is clear that a combination of direct mechanisms associated with the *C9ORF72*^RE^ mutation and downstream impacted cellular processes, including prominent neurophysiological perturbations, collectively contribute to *C9ORF72*^RE^ mediated-disease progression.

Neurophysiological dysfunction is established and prominent within the advanced stages of neurodegenerative disease patients where a complex combination of neuronal and synaptic loss in addition to neuronal dysfunction leads to a consensus systemic loss of function (Frere and Slutsky, 2018). However, in current years, the monitoring of non-symptomatic neurodegenerative patients, including *C9ORF72*^RE^ patients, carrying familial mutations is beginning to present a scenario whereby neurophysiological perturbations are evident before any notable clinical symptoms arise (Benussi et al., 2016; Geevasinga et al., 2016; Styr and Slutsky, 2018). Critically, these perturbations present as highly plausible, core contributors to disease pathogenesis, via neuronal injury through excitotoxicity and reduced function by way of impaired neurotransmission. Understanding the sources of the neurophysiological function and mechanisms directly linking these features to the molecular pathogenesis of the *C9ORF72*^RE^, thus have an important role to play in understanding ALS-FTD. Typically, we consider the general excitability of neurons to underpin its physiological function and is ultimately dependent upon a complex myriad of several factors including synaptic function, morphology and altered intrinsic excitability, which is dependent upon the functional expression of ion channels associated with action potential generation. This review summarizes the current literature describing *C9ORF72*^RE^-mediated neuronal dysfunction mechanisms in both cortical and motor neurons, contrasting these with each other as well as other ALS-FTD genetic backgrounds. We also review how these neurons may be impacted by other non-cell autonomous mechanisms involving glial cells. Finally, we will discuss our current understanding around the molecular determinants of this dysfunction and how these are linked to haploinsufficiency and related to repeat expansion toxic gain-of-function.

## Cortical Dysfunction in *C9ORF72* Repeat Expansion-Mediated Amyotrophic Lateral Sclerosis-Frontotemporal Dementia

**FIGURE 1:**
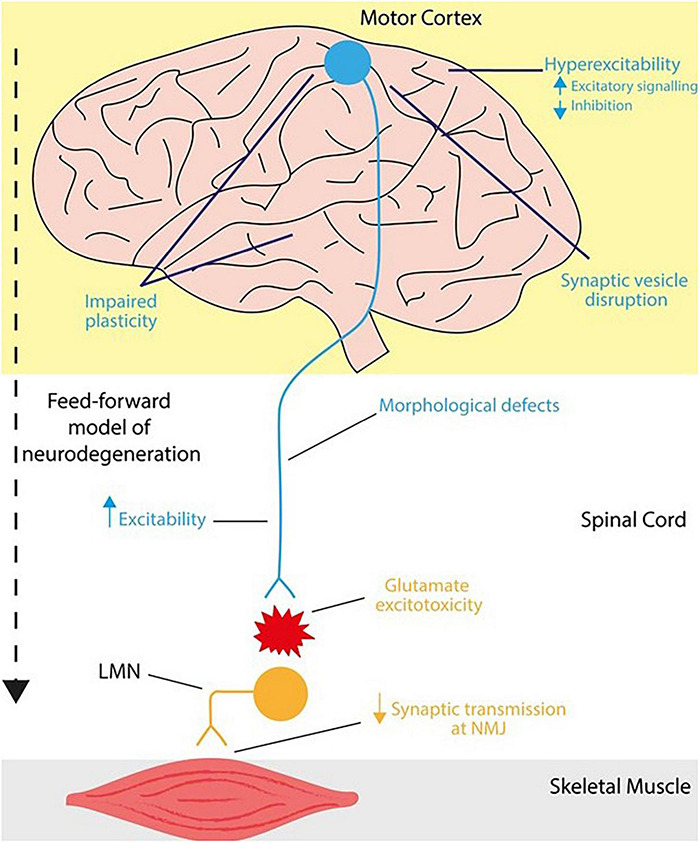
Mechanisms of neurophysiological impairments in the cortex and lower motor neurons in *C9ORF72*^RE^ ALS. In humans, upper motor neurons (*blue*) descend from the motor cortex and project onto the brainstem and spinal cord via the corticospinal tract. These corticospinal neurons form a monosynaptic pathway (in primates and humans) that innervates lower motor neurons (*orange*), which in turn transmit motor signals to effector muscles. Together this forms the motor circuitry within humans. Neurophysiological impairments leading to ALS can arise in the corticospinal tract at various loci. In ALS, cortical dysfunction ranges from hyperexcitability (increased excitability) as a result of increased excitatory signaling or reduced inhibition, disruption of synaptic vesicle dynamics and impaired synaptic plasticity that also extends to cortico-hippocampal connections. Within the corticospinal tract, upper motor neurons are vulnerable to synaptic loss and dendrite pathology including loss of dendritic spines that may arise from increased hyperexcitability. In a feedforward mechanism of dysfunction, degeneration of lower motor neurons is mediated, at least in part, via glutamate-mediated excitotoxicity whereby, cortical dysfunction precedes that of lower motor neurons, potentially causing further neurophysiological impairments and injury in lower motor neurons.

Beyond established degeneration of the motor cortex, neurophysiological disturbances in the cortex of ALS patients represents a longstanding pathological hallmark of disease. Such clinical observations are consistent between both sporadic and familial backgrounds (Geevasinga et al., 2016), including those harboring the *C9ORF72*^RE^ mutation (Williams et al., 2013; Benussi et al., 2016; Schanz et al., 2016; Nasseroleslami et al., 2019). Supported by extensive transcranial magnetic stimulation (TMS) (Vucic et al., 2013; Eisen et al., 2017) and resting state magnetoencephalography (MEG) studies (Proudfoot et al., 2016), cortical network dysfunction in ALS patients is found to manifest early, possibly prodromally, typically preceding lower motor neuron dysfunction leading to a possible staged continuum of pathogenesis consistent with a feed-forward mechanism of neurodegeneration (Geevasinga et al., 2016; Menon et al., 2017). Figure 1 summarizes this concept. Importantly cortical dysfunction is not limited to ALS, it is present in FTD patients (Lindau et al., 2003; Nishida et al., 2011) and is a common observation in other neurodegenerative diseases including Alzheimer’s, Parkinson’s and Huntington’s Disease (Palop et al., 2006; Styr and Slutsky, 2018; McColgan et al., 2020). Like for many other neurodegenerative diseases (Selkoe, 2002), functional synaptic perturbations at early disease stages are thought to drive cortical synaptic loss, which correlates with severe cognitive impairments observed in *C9ORF72*^RE^ patients (Henstridge et al., 2018). Further, magnetic resonance imaging (MRI) studies in ALS-FTD have demonstrated structural changes in the motor cortex that correlate with cognitive and behavioral impairments (Agosta et al., 2016; Consonni et al., 2018), in addition to functional defects that impact on cortical and subcortical activity (Mohammadi et al., 2015). Cortical dysfunction is therefore thought to play a key role in early pathogenic events in ALS-FTD. A summary of studies investigating *C9ORF72*^RE^ cortical dysfunction is presented in Table 1.

**TABLE 1:** Summary of physiological studies that have implication for the *C9ORF72*^RE^ in cortical dysfunction.

Cortical neurons	Study	Physiological finding	Link to *C9ORF72*^RE^	Method of investigation	Model
	Prudencio et al., 2015	Altered gene expression in synaptic signaling processes		Transcriptomics	*C9ORF72*^RE^ post-mortem material
	Henstridge et al., 2018	Decreased synaptic density associated with cognitive decline		Electron microscopy and array tomography	*C9ORF72*^RE^ post-mortem material
	Choi et al., 2019	Reduced synaptic (mEPSC) frequency	Poly-GR (80 repeat) DPRs	Patch-clamp electrophysiology (mEPSC recordings)	DPR mouse model
	Jensen et al., 2020	Reduced synaptic vesicle-associated protein 2 (SV2) Altered calcium homeostasis and impaired vesicle release	Poly-GA (149 repeat) DPRs	Calcium imaging FM dye vesicular imaging; Synaptic protein puncta	Primary rat cortical neurons *C9ORF72*^RE^ patient-derived iPSC cortical neurons
	Perkins et al., 2021	Increased network burst activity; increased synaptic density; impaired synaptic potentiation; reduced vesicular pool		Electrophysiology Transcriptomics; Synaptic staining	*C9ORF72*^RE^ patient-derived iPSC cortical neurons
	Barbier et al., 2021	Disease modifier mediated decrease of SV2 and synaptophysin		Immunostaining	*C9ORF72*^RE^ post-mortem cortex

*The table describes the main physiological findings of the study, whether they are associated with a mechanism of C9ORF72^RE^ pathology and upon which models these findings were obtained.*

### Network and Synaptic Plasticity

Functional nervous system plasticity presents the critical ability to modify neuronal properties in response to environmental demands and may manifest in a number of structural and functional changes that impact upon neurons and glial cells (Turrigiano, 2012; Suminaite et al., 2019). Functional impairments in plasticity are considered major early features of neurodegenerative disease and are representative of altered homeostasis that precedes and, potentially, drives further neuronal dysfunction and/or loss (Milnerwood and Raymond, 2010; Starr and Sattler, 2018; Styr and Slutsky, 2018). Synaptic plasticity is the process by which synapses undergo activity-dependent changes in their efficacy, where long-term potentiation (LTP), long-term depression (LTD), and spike-time dependent plasticity are the cellular correlates of, *inter alia*, cognitive processes such as learning and memory (Malenka and Bear, 2004). Paired associative stimulation using TMS techniques has revealed striking LTP–like network plasticity impairments in asymptomatic *C9ORF72*^RE^ patients indicative of early, widespread, cortical dysfunction of potential synaptic origins (Benussi et al., 2016). Benussi et al. (2016) predict that synaptic/network plasticity impairments present 15 years before symptomatic onset making these pathological observations some of the earliest evidenced in ALS-FTD patients. Direct evidence of impaired synaptic potentiation of mini excitatory post-synaptic currents was recently confirmed in induced pluripotent stem cell (iPSC)-derived cortical neurons generated from *C9ORF72*^RE^ patients, a feature that was rescued in isogenic, gene-corrected lines (Perkins et al., 2021). Beyond this, functional investigations of impaired synaptic plasticity in ALS and FTD have been determined in hippocampal murine preparations: *UBQLN2*^*P*497*H*^ (Gorrie et al., 2014); *SOD1*^*G*93*A*^ (Spalloni et al., 2011) and TDP-43 transgenic mice (Koza et al., 2019), TDP-43 conditional knockout mice (Wu et al., 2019). Further, impaired hippocampal synaptic plasticity is observed in non-TDP-43 FTD models [progranulin knock out mice (Petkau et al., 2012) and MAPT knock out mice (Ahmed et al., 2014; Biundo et al., 2018)]. Also, impaired plasticity has been observed at the neuromuscular junction of *Drosophila* over-expressing *C9ORF72*^RE^ (Perry et al., 2017). Broad cellular disruption affecting molecules and signaling processes relevant to synaptic plasticity are highlighted by transcriptional disturbances in both *C9ORF72*^RE^ patient-derived cortical neurons (Perkins et al., 2021) and *C9ORF72*^RE^ patient post-mortem cortex (Prudencio et al., 2015). Defined molecular pathological mechanisms of altered cortical synaptic plasticity in ALS-FTD remain to be elucidated. However, reduced LTP and LTD have been demonstrated in cortico-hippocampal connections of a murine *C9ORF72* knockout model, which highlight a role for the C9ORF72 protein in synaptic plasticity mechanisms in the hippocampus, but also potentially suggest that haploinsufficiency of the C9ORF72 protein may underpin some synaptic plasticity deficits (Ho et al., 2020). Indeed, the knockout of putative interactors with C9ORF72 also yields notable impairments in LTP (Gerges et al., 2004; Niu et al., 2020).

Plasticity has close association with homeostatic function and the inability to modify neuronal function in response to external stimuli suggests that plasticity impairments may represent very early markers of disease onset where cells are unable to respond to, as yet unknown, chronic disease-mediated changes (Milnerwood and Raymond, 2010; Benussi et al., 2016; Starr and Sattler, 2018; Styr and Slutsky, 2018). Future work upon impaired plasticity in ALS-FTD cortical neurons and circuitry promises to yield leads into the early drivers of disease.

### Cortical Hyperexcitability in Amyotrophic Lateral Sclerosis Patients Is a Hallmark Disease Feature

The neurophysiological profiling of ALS patients using transcranial magnetic stimulation has revealed considerable cortical and layer V projection neuron circuit perturbations that are consistent with a general increase in neuronal excitability within the motor cortex. Given these studies measure excitability from the motor cortex, early work did not show a correlation with (motor) cortical hyperexcitability being present in *C9ORF72*^RE^ FTD patients (Schanz et al., 2016), however, recent work has shown that increased strength of cortical hyperexcitability in ALS patients is associated with increased cognitive impairments (Agarwal et al., 2021). Nonetheless, cortical hyperexcitability has been observed in FTD models (García-Cabrero et al., 2013), but the degree to which it plays a pathogenic role in FTD is less understood. The observation of reduced short interval intracortical inhibition (SICI), first reported by Kujirai et al. (1993) in ALS patients, is highly consistent amongst sporadic and familial cases (Geevasinga et al., 2016), including *C9ORF72*^RE^ patients (Wainger and Cudkowicz, 2015; Schanz et al., 2016; Nasseroleslami et al., 2019), such that it is now considered a disease hallmark. Longitudinal assessments of ALS-FTD patients now indicate that reduced SICI manifests pre-symptomatically, preceding lower motor neuron dysfunction (Menon et al., 2015; Geevasinga et al., 2016) and becomes more pronounced with disease progression (Menon et al., 2020). Cortical circuits control upper motor neurons within the cortico-spinal tract, and hyperexcitability is associated with excitotoxicity, therefore cortical hyperexcitability is considered to be a pathogenic driver of motor neuron injury and dysfunction in ALS-FTD (Sahara Khademullah et al., 2020). Indeed, the degree of cortical hyperexcitability in ALS patients correlates with disease prognosis (Shibuya et al., 2016). Understanding the physiological and pathological determinants of cortical hyperexcitability in ALS-FTD is a key undertaking.

Physiological mechanisms explaining early cortical hyperexcitability are now emerging and center upon the deregulation of the complex synchronization of excitatory and inhibitory signaling within the cortex. Reduced cortical SICI argues toward a reduced inhibitory influence in the cortex of ALS-FTD patients. Accumulating evidence supports the involvement of inhibitory GABA-ergic interneurons, the predominant mediators of inhibitory activity in the networked circuitry of the cortex (Farrant and Nusser, 2005), as a pathological substrate in ALS-FTD patients. The loss of inhibitory signaling could be mediated via the loss of function or degeneration of interneurons thereby promoting cortical excitability. Recent work has demonstrated a reduction in parvalbumin interneurons, the major class of interneuron in the motor cortex (Estebanez et al., 2017), in a *TDP-43*^*Q*331*K*^ model of ALS-FTD and *C9ORF72*^RE^ ALS patient post-mortem suggesting the loss of inhibition may well also come from a selective vulnerability of this neuron class (Lin et al., 2021). In addition to this selective vulnerability, hippocampal interneurons appear to display considerable TDP-43 pathology in TDP-43 transgenic mice (Tsuiji et al., 2017). It may be therefore posited that cortical interneuron dysfunction is an early contributor to cortical hyperexcitability and that later interneuron degeneration contributes to a more pronounced hyperexcitability as the disease course progresses. However, to date there remains no data to assess the function of interneurons in the context of *C9ORF72*^RE^ and our understanding must therefore be derived via other genetic models of ALS. Selective pharmacological rescue of cortical interneuron function in a mutant *SOD1*^*G*93*A*^ mouse model preserves motor axon function and prolonged survival by rescuing reduced inhibitory input on to layer V projection neurons (Sahara Khademullah et al., 2020), suggesting that increasing interneuron function has the potential to reduce excitability in the motor cortex, thereby being neuroprotective to motor neurons. Although, we must also be careful in our assessment of long range impacts of cortical activity upon motor neuron function in ALS rodent models given that the monosynaptic cortico-spinal tract circuit is an anatomical feature that is exclusive to primates (Lemon, 2008). In contrast to the former study, Kim et al. revealed parvalbumin-expressing interneurons from neonatal and symptomatic *SOD1*^*G*93*A*^ mice had increased intrinsic excitability compared to wild-type interneurons (Kim et al., 2017). However, data from a *TDP-43*^*A*315*T*^ mouse model (Zhang et al., 2016) showed that young mice display sustained hyperexcitability in somatostatin-positive interneurons, but not in parvalbumin-positive neurons, which are hypoexcitable. Somatostatin interneurons regulate the excitability of parvalbumin interneurons, hence the hyperexcitability of somatostatin-expressing interneurons drives the hypoexcitable profile of parvalbumin interneurons, and in turn, causes hyperexcitability of the layer V projection neurons. In addition, recent studies have demonstrated that disturbances in the transcriptional landscape are consistent with an impact upon inhibitory synapses in *FUS*^Δ*NLS/+*^ murine model (Sahadevan et al., 2021; Scekic-Zahirovic et al., 2021). Importantly, the interneuronal hypothesis also extends to non-TDP-43 FTD forms where interneuronal pathology is observed in murine FTD models (Lui et al., 2016) and noting that interneurons control cortical network synchronicity, may underlie altered EEG activity in FTD patients (Lindau et al., 2003; Nishida et al., 2011). The underlying molecular pathological mechanisms of interneuronal dysfunction and loss in ALS-FTD remains to be determined.

Excitatory neurons represent approximately 80% of the adult cortex and numerous pieces of evidence are converging toward the contribution of these neurons to abnormalities in cortical excitability in ALS-FTD patients. Perkins et al. (2021) demonstrated that cultures of excitatory cortical neurons derived from *C9ORF72*^RE^ iPSCs displayed an enhanced network burst frequency compared to control derived neurons. These properties appear to be explained by the fact that *C9ORF72*^RE^ excitatory neurons had an increased functional synaptic input due to increased synaptic density, but not altered intrinsic excitability. Interestingly, an increased synaptic input of excitatory cortical neurons was seen in the motor cortex of pre-symptomatic (at postnatal day 30) mutant *TDP-43*^*Q*331*K*^ mice and *SOD*^*G*93*A*^ mice (Van Zundert et al., 2008; Fogarty et al., 2016a; Saba et al., 2016). Given that iPSC-derived cortical neurons are likely to reflect a physiologically early developmental status (Livesey et al., 2016) and that murine models show early disturbances, it is possible these studies are in line with an emerging consensus of increased excitatory synaptic activity as being a feature of cortical hyperexcitability. Importantly, this consensus may extend to other neurodegenerative diseases, such as Alzheimer’s Disease, where increased excitatory synaptic density and input early in disease is again observed in equivalent models (Šišková et al., 2014; Ghatak et al., 2019). Nonetheless, there are reports of excitatory input not changing in the *TDP-43*^*A*315*T*^ model (Zhang et al., 2016) and a *C9ORF72*^RE^ murine model, though this latter model does not display classical ALS-FTD pathology or neurodegeneration (Peters et al., 2015). Increased synaptic density in ALS-FTD is in clear contrast to the belief that neurodegeneration causes synaptic loss. Indeed, the degree of synaptic loss in the pre-frontal cortex of *C9ORF72*^RE^ ALS-FTD patient post-mortem correlates with the degree of cognitive impairment displayed by the patients (Henstridge et al., 2019). Furthermore, later stage, symptomatic P60 *TDP-43*^*A*315*T*^ mice exhibit layer V projection neurons with a decrease in synaptic input and spine density (Handley et al., 2017). These collective latter studies are therefore consistent with a trend that synaptic loss is restricted to latter stages of the disease course and accompanies the onset of symptomatic disease. The data would therefore suggest a shift from an early increased synaptic density property of *C9ORF72*^RE^ excitatory cortical neurons, supporting cortical hyperexcitability observations, to a general decrease, which appear to be aligned to symptomatic onset.

Mechanisms promoting cortical synaptic density remain unreported but are associated with transcriptional dysregulation consistent with modified expression of synaptic architecture proteins (Prudencio et al., 2015; Perkins et al., 2021). Conversely, mechanisms supporting synaptic loss in *C9ORF72*^RE^ cortical neurons are now emerging. Synaptic loss is observed in the prefrontal cortex of aged (4.5 months) transgenic mice expressing 80-repeat GR (GR_80_) DPRs (Choi et al., 2019). Furthermore, consistent with increasing reports of C9ORF72 localization at the synapse (Frick et al., 2018; Xiao et al., 2019), hippocampal regions of 3-month old C9ORF72 knockout mice show a reduction in synaptic density (Xiao et al., 2019), suggesting that haploinsufficiency may play a role in cortical synaptic loss. C9ORF72 is also highly expressed in microglia (Rizzu et al., 2016), and recent work has determined that loss of C9ORF72 exacerbates microglial synaptic pruning activity in the cortex, which correlates with cognitive impairments (Lall et al., 2021). Synaptic loss may therefore be driven by perturbed microglial function driven through C9ORF72 haploinsufficiency. Noting that microglia can equally sense and be regulated by neuronal excitability (Umpierre and Wu, 2021), how microglia contribute to cortical hyperexcitability or are potentially influenced by hyperexcitability will be a key question to resolve in how ALS-FTD progresses.

### Layer V Projection Neurons Display Hyperexcitability and Morphological Defects

Layer V cortical projection neurons (aka upper motor neurons) are a vulnerable population in ALS that functionally connect the motor cortex to lower motor neuron populations in the spinal cord. Reduced inhibition onto layer V neurons appears to drive hyperexcitability in this neuronal population in TDP-43^*A*135*T*^ mice (Zhang et al., 2016). Recent data from a rodent model in which hyperexcitability is chronically chemogenetically driven in upper motor neurons leads to the development of essential features of ALS, including upper and lower motor neuron degeneration, reactive gliosis and induced TDP-43 pathology (Haidar et al., 2021). Such data is consistent with the interrelation between hyperexcitability and the feed forward model of neurodegeneration. Furthermore, it appears that increased hyperexcitability can generate morphological changes. A study upon a nuclear localization sequence-deficient mouse model of TDP-43 identified that cytoplasmic mislocalization of TDP-43 drives intrinsic hyperexcitability and decreased excitatory synaptic inputs (Dyer et al., 2021). Indeed, hyperexcitability may drive continued functional synaptic loss, dendritic spine loss and dendrite pathology in upper motor neurons that are commonly observed features in upper motor neurons of ALS patient post-mortem tissue (Hammer et al., 1979; Genç et al., 2017) and other models, including *TDP-43*^*A*315*T*^ (Handley et al., 2017), *SOD1*^*G*93*A*^ (Fogarty et al., 2016b,^2017^), and *FUS*^*R*521*G*^ (Sephtona et al., 2014). Clearly, data on *C9ORF72*^RE^ remains scant for this cell type. Future work will be required to clarify whether impairments in layer V projection neurons are determined by intrinsic, cell autonomous mechanisms and/or are driven by altered input via cortical dysfunction which in turn drives hyperexcitability and synaptic loss.

### At What Point Does the Cortex Become Dysfunctional in *C9ORF72*^RE^ Amyotrophic Lateral Sclerosis-Frontotemporal Dementia?

Disease progression in neurodegenerative diseases is thought to reflect a stage of homeostatic adaptation, where disease-driven disturbances in network function are functionally tolerated for an undetermined period of time, but ultimately give way to network failure, where homeostasis mechanisms cannot viably maintain function (Frere and Slutsky, 2018). There is potential evidence for functional changes in ALS-FTD models that may reflect this early shifting landscape. Longitudinal assessment of synaptic and intrinsic excitability of *SOD1*^*G*93*A*^ layer V cortical projections reveal a fluctuating reciprocal profile of altered intrinsic excitability and synaptic input that could reflect functional adaptation at the pre-symptomatic stage (Kim et al., 2017). Similarly, despite *C9ORF72*^RE^ iPSC-derived cortical neurons exhibiting increased synaptic density, consistent with increased excitability (and burst frequency), neurons also display a reduced readily releasable pool of synaptic vesicles. As a result, these neurons display a reduced ability to maintain synaptic transmission and present a reduced burst duration (Perkins et al., 2021). These latter data are consistent with a putative role of C9ORF72 protein in vesicular trafficking within the *trans*-Golgi network (Snowden et al., 2012; Aoki et al., 2017; Frick et al., 2018) and that C9ORF72 haploinsufficiency may result in a reduction of the readily releasable pool of vesicles. Consistent with these data, mice engineered to express 149-repeat GA (GA_149_) DPRs also demonstrate a reduced expression of synaptic vesicle protein SV2 in addition to altered Ca^2+^ homeostasis and impaired vesicle release in cortical neurons (Jensen et al., 2020). In this regard, such reductions in general vesicular function may potentially reflect early homeostatic adaptations in response to increased synaptic density (or vice versa) driven by pathological *C9ORF72*^RE^-related mechanisms. Interestingly, potential modifiers of disease onset in *C9ORF72*^RE^ FTD-mediated disease appear to be associated with altered expression of proteins with synaptic roles including synaptic vesicle dynamics (Barbier et al., 2021). Given that cortical function can be readily monitored in patients, establishing the earliest known physiological disturbances in cortical function in *C9ORF72*^RE^ and the pathological drivers underpinning this may give us one of our earliest windows into understanding *C9ORF72*^RE^ disease onset and progression.

## Lower Motor Neuron Dysfunction in *C9ORF72*^RE^-Mediated Amyotrophic Lateral Sclerosis- Frontotemporal Dementia

In receiving monosynaptic innervation from upper motor neurons, lower spinal motor neurons represent the final effector component of the lower motor system, whose axons project to innervate skeletal muscle fibers (Burke, 1981). Lower motor neuron dysfunction has long been an established clinical observation, detected by nerve conduction and electromyography (EMG) and are key criteria in the diagnosis of ALS (Daube, 1985; Mogyoros et al., 1998; Geevasinga et al., 2015; de Carvalho and Swash, 2016) including *C9ORF72*^RE^ patients (Geevasinga et al., 2015). Critically, altered lower motor neuron function in ALS patients is evidenced after that of cortical dysfunction and parallels the onset of patients developing muscle weakness, atrophy, fasciculation and cramps in ALS (Menon et al., 2015). Further, the development of fasciculation and cramps precede that of muscle weakness suggesting that hyperexcitability leading to progressive loss of function (hypoexcitability) is a feature of the lower motor neuron in ALS disease progression before eventual degeneration and loss (Bae et al., 2013). In this aspect of the review, we will discuss the mechanisms leading to the potential changes in excitability and neurophysiological mechanisms leading to excitotoxicity and cell death. These studies are summarized in Table 2.

**TABLE 2:** Summary of physiological studies that have implication for the *C9ORF72*^RE^ in lower motor neuron dysfunction.

Lower motor neurons	Study	Physiological finding	Link to C9ORF72^RE^	Method of investigation	Model
	Donnelly et al., 2013	Increased susceptibility to glutamate-mediated excitotoxicity	Toxic RNA gain-of-function	Transcriptomics; Excitotoxicity assay	*C9ORF72*^RE^ patient-derived iPSC motor neurons
	Sareen et al., 2013	Intrinsic hypoexcitability		Patch-clamp electrophysiology	*C9ORF72*^RE^ patient-derived iPSC motor neurons
	Wainger et al., 2014	Hyperexcitability and pharmacological rescue using Kv7 channel activator		Multielectrode array and patch-clamp electrophysiology; Pharmacological rescue	*C9ORF72*^RE^ patient-derived iPSC motor neurons
	Devlin et al., 2015	Initial intrinsic hyperexcitability, followed by hypoexcitability and loss of synaptic activity		Patch-clamp electrophysiology	*C9ORF72*^RE^ patient-derived iPSC motor neurons
	Zhang et al., 2015	Impaired synaptic release at the neuromuscular junction	Disruption of normal RNA processing	Patch-clamp electrophysiology	*C9ORF72*^RE^ patient-derived iPSC motor neurons
		Reduction in active zones number			(C_4_G_2_)_30_ *Drosophila* model
	Freibaum et al., 2015	Impaired synaptic release at the neuromuscular junction	Disruption of normal RNA processing	Neuronal phenotype analysis	(C_4_G_2_)_58_ *Drosophila* model
		Reduction in active zones number			
	Perry et al., 2017	Impaired synaptic plasticity at the neuromuscular junction	Poly-GR (100 repeat) DPRs	Patch-clamp electrophysiology	*C9ORF72*^RE^ *Drosophila* model
	Xu and Xu, 2018	Increased extracellular glutamate and intracellular Ca^2+^ levels	Poly-GR/PR (36 repeat) DPRs	Glutamate and calcium imaging	*C9ORF72*^RE^ *Drosophila* model
		Increased in NMDA receptor mediated signaling			
		Increase of synaptic boutons and active zones in larval neuromuscular junctions			
	Shi et al., 2018	Increased susceptibility to glutamate-mediated excitotoxicity	C9ORF72 haploinsufficiency	Excitotoxicity; Pharmacological rescue; Immunostaining; Biochemical (Western blot, qPCR)	*C9ORF72*^RE^ patient-derived iPSC motor neurons
		Increased functional expression of calcium permeable AMPAR			
		Upregulation of NMDA receptor subunit GluN1			
	Selvaraj et al., 2018	Increased susceptibility to glutamate-mediated excitotoxicity		Patch-clamp electrophysiology; BaseScope Assay; Biochemical (Western blot, qPCR)	*C9ORF72*^RE^ patient-derived iPSC motor neurons *C9ORF72*^RE^ post-mortem material
		Increased GluA1 AMPA subunit expression			
		Increased functional expression of calcium permeable AMPAR			
	Bursch et al., 2019	Increased susceptibility to glutamate-mediated excitotoxicity		Calcium imaging	*C9ORF72*^RE^ patient-derived iPSC motor neurons
	Gregory et al., 2020	Increased GluA1 AMPA subunit expression selective to motor neurons, not cortical neurons		BaseScope	*C9ORF72*^RE^ patient post-mortem material
	Zhao et al., 2020	Astrocyte-mediated hypoexcitability		Patch-clamp electrophysiology; Transcriptomics	*C9ORF72*^RE^ mutant iPSC-derived astrocytes
	Catanese et al., 2021	Hypoexcitability driven loss of excitatory synapses through a CREB-dependent signaling pathway		Transcriptomics; Proteomics; Optogenetics Pharamcological rescue	*C9ORF72*^RE^ patient-derived iPSC motor neurons
	Castelli et al., 2021	Manipulating SK ion channel activity improves C9ORF72-ALS motor neuron death and Drosophila locomotor deficits		Transcriptomics; Pharmacological rescue	*C9ORF72*^RE^ patient-derived iPSC motor neurons
					*C9ORF72*^RE^ *Drosophila* model
	Butti et al., 2021	Reduction in the number of presynaptic and postsynaptic structures at the NMJ	C9ORF72 haploinsufficiency	Patch clamp electrophysiology;	*C9ORF72*^RE^-related zebrafish
				Mass spectrometry	

*The table describes the main physiological findings of the study, whether they are associated with a mechanism of C9ORF72^RE^ pathology and upon which models these findings were obtained.*

### Loss of Synaptic Innervation From Upper Motor Neurons

Synaptic glutamatergic signaling links upper and lower motor neuron function, and, glutamate-mediated excitotoxicity is considered one of the main pathogenic mechanisms that contributes to the degeneration of motor neurons in ALS-FTD (Cleveland and Rothstein, 2001). The hypothesis is multifaceted and includes cell autonomous and non-cell autonomous mechanisms. Commensurate with cortical disease progression, synaptic loss in lower motor neurons is an established observation in the latter stages of ALS and is expected to be a major physiological determinant preventing lower motor neuron function in the later stages of disease (Sasaki and Maruyama, 1994). Synaptic loss accompanied by CREB-dependent transcriptomic and proteomic changes is observed in *C9ORF72*^RE^ iPSC-derived motor neurons maintained for extended culture time (Catanese et al., 2021). A number of hypotheses center upon disturbances in glutamate-mediated signaling and altered excitability being major contributors to synaptic loss and other sites of lower motor neuron injury.

Lower motor neurons are responsive to synaptic glutamate via the synaptic expression of glutamatergic AMPA and NMDA receptors (Van Den Bosch et al., 2006). Early work determined an apparent intrinsic vulnerability of lower motor neurons to AMPA receptor-mediated excitotoxicity (Rothstein et al., 1990, 1992; Couratier et al., 1993; Rothstein, 1995; Cleveland and Rothstein, 2001). Elevated synaptic glutamate levels are predicted given the increased excitability of upper motor neurons. However, whether pre-synaptic terminal deficits in glutamate release from upper motor neurons exist remains to be determined. Nonetheless, the uptake of glutamate from the synaptic cleft is strongly hypothesized to be reduced given that the expression of astrocyte glutamate transporter (EAAT2) is widely reported to be attenuated in multiple ALS models (Rosenblum and Trotti, 2017). In the case of *C9ORF72*^RE^ however, patient-derived astrocytes are not consistent with a reduction in EAAT2 expression or function (Allen et al., 2019b; Zhao et al., 2020). How such data are to be reconciled with other ALS models of glutamate transporter dysfunction and expression remains to be resolved.

Over stimulation of glutamate receptors gives rise to the possibility of an injurious, excitotoxic level of Ca^2+^ influx (Pina-Crespo et al., 2014) and iPSC-derived motor neurons obtained from *C9ORF72*^RE^ patients exhibit enhanced vulnerability to glutamate receptor-mediated excitotoxicity (Donnelly et al., 2013; Selvaraj et al., 2018; Shi et al., 2018; Bursch et al., 2019). Interestingly, this vulnerability has been rescued pharmacologically in studies using an anticoagulation-deficient form of activated protein C (Shi et al., 2019) and antisense oligonucleotides against the repeat expansion (Donnelly et al., 2013). Mechanistically, this vulnerability has been shown to occur as a result of increased relative expression of Ca^2+^ permeable AMPA receptors in *C9ORF72*^RE^ patient-derived motor neurons due to a greater expression of Ca^2+^ permeable AMPA receptor subunit GluA1 (Selvaraj et al., 2018; Shi et al., 2018; but see Moore et al., 2019). Further work on *C9ORF72*^RE^ patient post-mortem demonstrated that the dysregulation of GluA1 is selective to *C9ORF72*^RE^ lower motor neurons and is not present in the cortex, and thus providing an example of a regional specific degenerative mechanism (Selvaraj et al., 2018; Gregory et al., 2020). The dysregulation of GluA1 also appears conserved amongst other genetic ALS backgrounds including mutant TDP-43 motor neurons (Bursch et al., 2019), FUS (Udagawa et al., 2015) and in sporadic ALS patients, where the latter show further dysregulation of AMPA receptor subunits in the cortex (Gregory et al., 2020). In agreement, data from mSOD1 patients and models indicate a converging mechanism of vulnerability to glutamate-mediated excitotoxicity via Ca^2+^ permeable AMPA receptors (Shaw, 2005; Van Den Bosch et al., 2006), where such studies appear consistent with a reduction in the relative expression of GluA2 subunits, the master regulators of Ca^2+^ permeability. The GluA2 subunit achieves this because it predominantly presents in its post-transcriptionally edited form where a channel-lining, positively charged arginine side chain protrudes into the ion channel, presenting a charge block to Ca^2+^ flux (Traynelis et al., 2010) whereas, the pre-edited GluA2 form contains a non-charged glutamine side chain and permits Ca^2+^ flux. Notably, inefficient RNA editing of the GluA2 subunit, thus resulting in Ca^2+^-permeability, has been reported in sporadic ALS patient samples (Kawahara et al., 2004a,b). However, whilst appearing to impact upon the function of editing enzyme ADAR2, this mechanism does not appear to be the source of increased Ca^2+^-permeable AMPA receptors in *C9ORF72*^RE^ patients (Selvaraj et al., 2018; Moore et al., 2019). Mechanisms of GluA1 upregulation in the context of *C9ORF72*^RE^ has been associated with haploinsufficiency of C9ORF72 protein in lower motor neurons derived from patient iPSCs and C9ORF72 knockout mice (Shi et al., 2018) and also hippocampal neurons (Xiao et al., 2019). In support of this, the knockout of putative C9ORF72 interactor Rab39b in primary neuron culture results in increased GluA1 trafficking to dendrites (Mignogna et al., 2015, 2021). Interestingly, despite early work indicating the low impact of NMDA receptor-mediated excitotoxicity on motor neurons, recent studies have shown an upregulation of the NMDA receptor subunit GluN1 (Shi et al., 2018) that can be rescued along with GluA1 upregulation using small molecule inhibitors of phosphatidylinositol-5-kinase signaling (Staats et al., 2019). The role of NMDA receptors in glutamate-mediated excitotoxicity and the role of DPRs remains to be fully explored. However, recent *Drosophila* models presenting poly-GR and PR (GR_36_, PR_36_) constructs demonstrated an increase in NMDA receptor-mediated signaling in glutamatergic neurons, suggesting that DPRs may have a role in the dysregulation of glutamate receptors in *C9ORF72*^RE^ motor neurons (Xu and Xu, 2018).

Clearly, a strong emphasis of research thus far has been given to the perturbations associated with glutamatergic signaling. The disruption of inhibitory GABA-ergic and glycinergic signaling in the spinal tract has been implicated in ALS, largely in mSOD1 models (Martin and Chang, 2012), though initial reports indicated this was a secondary event to motor neuron degeneration (Hossaini et al., 2011). Though, more recent work in *SOD1*^*G*93*A*^ mice now implicates deficits in inhibitory signaling associated with V1 interneurons in the spinal tract that parallel motor disturbances, raising the possibility that increased excitatory signaling in ALS patients may also stem from a reduced impact of inhibitory influences (Allodi et al., 2021). The specific impact of the *C9ORF72*^RE^ mutation on inhibitory signaling whether in the cortex or lower motor neuron remains to be determined.

### Altered Excitability in Lower Motor Neurons

Altered motor neuron excitability in *C9ORF72*^RE^ patients is predicted to be underpinned by changes in intrinsic expression of ion channels that support action potential conduction (Geevasinga et al., 2015). Physiological mechanisms addressing lower motor neuron excitability in the context of *C9ORF72*^RE^ have been widely investigated *in vitro*, employing iPSC-derived motor neurons from *C9ORF72*^RE^ ALS patient fibroblasts. Patch-clamp studies have reported hyperexcitability at early stages of motor neuron differentiation (2–6 weeks in culture) where cells become intrinsically more excitable to depolarization (Devlin et al., 2015; Wainger and Cudkowicz, 2015). However, as cultures are maintained further (7–10 weeks), they become hypoexcitable, evidenced by a reduction in action potential generation in response to depolarization compared to motor neurons derived from healthy patients (Sareen et al., 2013; Zhang et al., 2013; Devlin et al., 2015; Naujock et al., 2016; Guo et al., 2017). No changes in cell survival were reported in these studies, which supports the idea that changes in excitability are early signs of functional loss of motor neurons prior to their degeneration, which is also supported by clinical studies of motor function in ALS patients (Iwai et al., 2016). Furthermore, these data are broadly consistent with the overall consensus nature of shifting excitability in mutant SOD1 mice motor neurons that display a period of early hyperexcitability before hypoexcitability (Leroy and Zytnicki, 2015), which in turn precedes motor neuron denervation (Martínez-Silva et al., 2018).

Key mechanisms that drive these excitability states are now emerging. Selective tuning of cortical inhibition in *SOD1*^*G*93*A*^ mice to reduce potential cortical hyperexcitability has a protective impact upon lower motor neurons (Sahara Khademullah et al., 2020), suggesting that early hyperexcitability in lower motor neurons, at least in part, is driven by upstream cortical and upper motor neuron dysfunction and may drive injury or further pathological processes. On this note, increased depolarization of motor neurons, including via glutamate, has the ability to promote the formation of TDP-43 pathology (Weskamp et al., 2020) and drive DPR formation (Westergard et al., 2019). Interestingly, more recent data using improved iPSC-derived MN protocols yielding enriched, predominant neuronal cultures with very little glial differentiation does not exhibit any consistent differences in motor neuron excitability (Selvaraj et al., 2018; Zhao et al., 2020). This discrepancy from previous studies appears to be resolved by the fact that co-cultures of motor neurons with *C9ORF72*^RE^ astrocytes are induced to be hypoexcitable and that previous studies used protocols with heterogeneous cellular specification including astrocytes (Zhao et al., 2020). Beyond other impacts upon motor neuron health (Serio et al., 2013; Meyer et al., 2014; Allen et al., 2019a), the roles of astrocytes are therefore likely to play critical non-cell autonomous roles in the modulation of motor neuron excitability. This mechanism may be related to a soluble transmissible factor given that cultures of murine motor neurons with conditioned medium derived from *SOD1*^*G*93*A*^ expressing astrocytes, was found to alter ion channel function and motor neuron excitability (Fritz et al., 2013). Furthermore, recent data implicate a decreased expression of astrocyte K_*IR*_4.1-containing ion channels to adequately remove potassium extruded from active motor neuron axons in mSOD1 models indicating possible mechanisms impacting the ability of astrocytes to adequately maintain axonal homeostasis (Kelley et al., 2018). It therefore appears that numerous cell autonomous and non-autonomous mechanisms are at play here and not limited to intrinsic lower motor neuron processes.

The rescue of both hypoexcitability and hyperexcitability in motor neurons has been a pharmacological target in recent years. The promotion of increased excitability via pharmacological inhibition of small conductance calcium-activated potassium (SK) channels promotes survival and restores the activity-dependent transcriptional profiles and synaptic composition in *C9ORF72*^RE^ iPSC-derived motor neurons, and furthermore, promotes locomotor function in a *Drosophila* model containing 36 hexanucleotide repeats (Castelli et al., 2021; Catanese et al., 2021). *C9ORF72*^RE^ motor neurons also demonstrated an increase in the expression of SK channel subunits, which could be corrected using specific inhibition of the SRSF1-dependent nuclear export of pathological *C9ORF72*^RE^ transcripts (Castelli et al., 2021). Contrastingly, hyperexcitability in lower motor neurons has been established in several other ALS models and studies have used pharmacological activators of Kv7 potassium ion channels to reduce hyperexcitability in *C9ORF72*^RE^-derived motor neurons with the possibility that they protect motor neurons from excitotoxicity (Wainger et al., 2014; Huang et al., 2021). These studies have now been translated into clinical trials (Wainger et al., 2021). Our current understanding of the shifting excitability in both cortical and motor neurons indicates that the pharmacological benefit of modulators of excitability to patients will need to be understood and carefully considered according to disease stage.

### Impaired Neurotransmitter Release Is a Feature of the Neuromuscular Junction in Amyotrophic Lateral Sclerosis

Motor neuron denervation from the neuromuscular junction precedes motor neuron loss. Given that motor activity is required for the maintenance of innervation, it is no surprise that loss of motor neuron pre-synaptic activity is associated with disease pathogenesis. Measurable loss of motor input is common to symptomatic ALS patients and consistent with pre-synaptic dysfunction of motor neurons (Maselli et al., 1993) that is observed primarily, and more aggressively, in association with neuromuscular junctions innervated by fast-twitch motor neurons in *SOD1*^*G*93*A*^ mice (Cappello and Francolini, 2017). Beyond rodent models, reduced synaptic function has now been observed in several other models including *Drosophila* and zebrafish (Cappello and Francolini, 2017; Butti et al., 2021). Studies in fly models overexpressing hexanucleotide repeats (58 and 30 repeats) demonstrate impaired synaptic release at the neuromuscular junction and a reduced number of active zones in motor neurons (Freibaum et al., 2015; Zhang et al., 2015). Consistent with a reduction in synaptic activity over time, spontaneous post-synaptic current activity was shown to progressively decrease in *C9ORF72*^RE^ iPSC-derived motor neurons and was directly associated with hypoexcitability, but not motor neuron loss (Devlin et al., 2015). The potential for impaired synaptic release may be therefore related to an inherent inability to generate sufficient action potential activity at the pre-synaptic terminal. Moreover, Jensen and colleagues recently reported aberrations in vesicle dynamics that coincide with the loss of vesicle protein SV2 and precede motor neuron loss in a poly-GA (GA_149_) animal model and *C9ORF72*^RE^ motor neurons (Jensen et al., 2020). Interestingly, such observations align with investigations in cortical neurons and suggests that not only do poly-GA repeats interfere with the synaptic release mechanism but also the reduction in vesicular dynamics in the cortex may have mechanistic overlap with motor neurons (Jensen et al., 2020). This study also reports that altered vesicle dynamics are associated with elevated Ca^2+^ influx, which controls synaptic vesicular release (Jensen et al., 2020). It has therefore been hypothesized that the increased cytoplasmic Ca^2+^ may form a homeostatic mechanism to potentially rescue synaptic release. Indeed, the pharmacological rescue of *C9ORF72*^RE^ model (GR_100_) via the induction of endogenous NMJ plasticity signaling can rescue synaptic function (Perry et al., 2017). Moreover, Coyne et al. report that synaptic vesicle cycling defects due to deficits in the post-transcriptional inhibition of Hsc70-4/HSPA8 expression are common to *C9ORF72*^RE^ and mTDP-43 *Drosophila* models (Coyne et al., 2017), suggesting that vesicle depletion is at play at the NMJ. Importantly, this mechanism is linked to dynamin function, a key player in axonal transport, and therefore suggests that synaptic vesicle impairments and established impairments in axonal transport in ALS are potentially linked (Gunes et al., 2020).

## Summary

Our review provides an overview of the key concepts of neurophysiological disturbances in *C9ORF72*^RE^-mediated ALS-FTD. We have provided details on the current mechanistic view of the sources of these perturbations, when these appear in disease and allude to their relevance to pathogenesis. Many aspects of neurophysiological dysfunction in the context of *C9ORF72*^RE^-mediated disease are currently inferred. In this respect, a general consensus of early cortical hyperexcitability progressing to general loss of function consistent with hypoexcitability in the symptomatic period appears to be consistent across patients and, importantly, several ALS-FTD models appear to replicate this progression, at least in aspects (summarized in Table 3). However, there are established examples of mechanistic disturbances that differ from other genetic backgrounds. Similarly, pathogenesis of cortical and motor dysfunction display overlapping dysfunctional features but also selective regional differences.

**TABLE 3:** Summary of the main physiological observations that are associated with ALS.

Physiological observation	Evidence in non-*C9ORF72*^RE^ ALS-FTD	Evidence in *C9ORF72*^RE^ ALS-FTD
**Patient cortical hyperexcitability**	Established as a hallmark observation in ALS, including sporadic (reviewed Geevasinga et al., 2016). Evidenced pre-symptomatically (Geevasinga et al., 2016; Menon et al., 2017), and prominence increases with disease onset (Menon et al., 2020). Evidenced in FTD patients (Lindau et al., 2003; Nishida et al., 2011) and associated with cognitive decline.	Motor cortical hyperexcitability evidenced in *C9ORF72*^RE^ ALS (Williams et al., 2013; Benussi et al., 2016; Schanz et al., 2016; Nasseroleslami et al., 2019). Increased strength of cortical hyperexcitability observed in ALS-FTD patients correlates with increased cognitive impairments (Agarwal et al., 2021).

**Altered neuronal plasticity**	Hippocampal synaptic plasticity was evidenced in murine models: *UBQLN2*^*P*497*H*^(Gorrie et al., 2014); *SOD1*^*G*93*A*^ (Spalloni et al., 2011); TDP-43 transgenic mice (Koza et al., 2019), TDP-43 conditional knockout mice (Wu et al., 2019); non-TDP-43 FTD models (progranulin knock out mice (Petkau et al., 2012) and MAPT knockout (Ahmed et al., 2014; Biundo et al., 2018).	Patient synaptic/network plasticity observations are present in presymptomatic disease stages (Benussi et al., 2016). Synaptic plasticity defects are highlighted in iPSC-derived *C9ORF72*^RE^ cortical neurons (Perkins et al., 2021) and *C9ORF72*^RE^ postmortem cortex (Prudencio et al., 2015). Impaired plasticity at the neuromuscular junction of *C9ORF72*^RE^ *Drosophila* (Perry et al., 2017).

**Presymptomatic changes in cortical neurophysiological function**	Increase in synaptic input and intrinsic excitability in murine models of SOD1^*G*93*A*^ (Kim et al., 2017). Reduction in cortical interneurons in *TDP-43*^*Q*331*K*^ knock-in mouse (Lin et al., 2021). Cortical hyperexcitability observed in FTDP-17 mouse model of FTD (García-Cabrero et al., 2013).	Elevated network burst activity and enhanced synaptic input was found in iPSC-derived *C9ORF72*^RE^ cortical neurons, linked with decreased synaptic density, but not altered intrinsic excitability (Perkins et al., 2021).
	*TDP*^*A*315*T*^ mouse model show sustained hyperexcitability in somatostatin-positive interneurons, but hypoexcitability in parvalbumin-positive neurons (Zhang et al., 2016).	
	Increased synaptic input of excitatory cortical neurons was evidenced in the motor cortex of pre-symptomatic mutant *TDP-43*^*Q*331*K*^ mice and *SOD*^*G*93*A*^ mice (Van Zundert et al., 2008; Fogarty et al., 2016a; Saba et al., 2016). Presymptomatic excitability changes in ALS models reviewed in Gunes et al., 2020.	

**Post-symptomatic cortical neurophysiological function**	Decreased in synaptic input in ALS post-mortem tissue (Hammer et al., 1979; Genç et al., 2017) and other models, including *TDP-43*^*A*315*T*^ (Handley et al., 2017), *SOD1*^*G*93*A*^ (Fogarty et al., 2016b,^2017^), and *FUS*^*R*521*G*^ (Sephtona et al., 2014).	Synaptic loss was found to correlate with cognitive decline (Henstridge et al., 2018; Lall et al., 2021). Synaptic loss was observed in the prefrontal cortex of aged (4.5 months) transgenic mice expressing 80-repeat GR (GR_80_) DPRs (Choi et al., 2019).
	TDP-43 mouse model shows intrinsic hyperexcitability and decreased excitatory synaptic inputs (Dyer et al., 2021).	Hippocampal regions of 3-month-old *C9ORF72* knockout mice show a reduction in synaptic density (Xiao et al., 2019).
	Symptomatic *TDP-43*^*A*315*T*^ mice exhibit layer V projection neurons with a decrease in synaptic input and spine density (Handley et al., 2017). Post-symptomatic excitability changes in ALS models reviewed in Gunes et al. (2020).	

**Patient lower motor neuron excitability**	Axonal hyperexcitability and decreased function with symptomatic onset was reported in sporadic ALS patients (Geevasinga et al., 2015). Increased motor unit excitability, increased presence of fasciculation potentials, single unit motor unit firing, increased axonal excitability (reviewed in Gunes et al., 2020).	Increased axonal excitability has been highlighted in symptomatic *C9ORF72*^RE^ ALS patients (Geevasinga et al., 2015).

**Models of lower motor neuron intrinsic excitability**	Evidence of early hyperexcitability was seen in mSOD1 models (reviewed in Gunes et al., 2020).	Evidence of hyperexcitability at early stages of motor neuron differentiation (Devlin et al., 2015; Wainger and Cudkowicz, 2015) switching to hypoexcitability with culture time (Sareen et al., 2013; Devlin et al., 2015; Zhang et al., 2015; Naujock et al., 2016; Guo et al., 2017).
	Shifting excitability in mutant SOD1 mice motor neurons that display a period of early hyperexcitability before hypoexcitability (Leroy and Zytnicki, 2015) preceding motor neuron denervation (Martínez-Silva et al., 2018). *SOD1*^*G*93*A*^ expressing astrocytes was found to alter ion channel function and motor neuron excitability (Fritz et al., 2013).	Increased excitability via pharmacological inhibition of small conductance calcium-activated potassium (SK) channels promotes survival and restores the activity-dependent transcriptional profiles and synaptic composition in iPSC-derived *C9ORF72*^RE^ motor neurons, and furthermore, promotes locomotor function in a Drosophila model containing 36 hexanucleotide repeats (Castelli et al., 2021; Catanese et al., 2021).

**Loss of motor neuron synaptic input**	Synaptic changes in ALS models reviewed in Gunes et al. (2020).	Decreased synaptic activity and spontaneous post-synaptic current activity was evidenced in iPSC-derived *C9ORF72*^RE^ motor neurons (Devlin et al., 2015).

**Loss of NMJ function and innervation**	mTDP-43 *Drosophila* shows synaptic vesicle cycling defects (Coyne et al., 2017).	*C9ORF72*^RE^ Drosophila over-expressing hexanucleotide repeats (58- and 30-repeats) exhibit impaired synaptic release at the neuromuscular junction and decrease in number of active zones (Freibaum et al., 2015; Zhang et al., 2015). Decreased synaptic arborization and active zone number at neuromuscular junction in *C9ORF72*^RE^ patient-derived motor neurons (Perry et al., 2017). Impaired vesicle dynamics that precede motor neuron loss have been evidenced in GA mouse model and *C9ORF72*^RE^ patient-derived motor neurons (Jensen et al., 2020).

**Glutamate excitability**	mSOD1 patients and models exhibit vulnerability to glutamate-mediated excitotoxicity (Shaw, 2005; Van Den Bosch et al., 2006).	*C9ORF72*^RE^ patient-derived iPSC motor neurons exhibit enhanced vulnerability to glutamate receptor-mediated excitotoxicity (Donnelly et al., 2013; Selvaraj et al., 2018; Shi et al., 2018; Bursch et al., 2019). *C9ORF72*^RE^ post-mortem demonstrated that the dysregulation of GluA1 is selective to *C9ORF72*^RE^ lower motor neurons and is not present in the cortex (Selvaraj et al., 2018; Gregory et al., 2020).
	GluA1 dysregulation is evidenced in mutant TDP-43 motor neurons (Bursch et al., 2019), FUS (Udagawa et al., 2015) and in sporadic ALS patients (Gregory et al., 2020). Inefficient RNA editing of GluA2 subunits in sporadic ALS patients (Kawahara et al., 2004a).	

*The table details the prominent pathophysiological concepts that are thought to play a role in the pathogenesis of ALS; for example, cortical hyperexcitability and glutamate dysfunction in lower motor neurons. We summarize papers that provide data in non-C9ORF72^RE^ models and contrast these in current C9ORF72^RE^ models.*

Current models of *C9ORF72*^RE^ are broad, and are known to have both advantages and disadvantages especially in regards to their inability to fully capture the disease phenotype (Sances et al., 2016; Balendra and Isaacs, 2018). New improvements in disease modeling are needed to forward our understanding of disease pathogenesis and progress is now being made in this respect. For example, the ability to model the cortico-spinal tract *in vitro* in a human context is now documented (Andersen et al., 2020) and furthermore, it is now possible to examine the impact of native length DPR species in *Drosophila* (West et al., 2020). Importantly, the development of *C9ORF72*^RE^ rodent models that successfully recapitulate major aspects of ALS-FTD remains ongoing (Balendra and Isaacs, 2018). Such tools will allow us to systematically define the mechanistic impact of the *C9ORF72*^RE^ on cell types, as well as cell types upon each other.

In this regard, neurophysiological disturbances in ALS-FTD are now much more complex than previously believed. Beyond multiple molecular mechanisms associated with the *C9ORF72*^RE^, disturbances are likely to have an additional non-cell autonomous component relating to other dysfunctional cell-types that now include astrocytes and microglia. It is also becoming clear that for all neurodegenerative disease early functional changes may partially reflect homeostatic mechanisms that counteract disease-driven pathophysiology. On this note, emerging work is now beginning to consider the cortico-spinal circuit as a singular functional unit and this work will allow us to identify how each component can potentially impact each other. Such data will help stratify early mechanisms associated with disease progression for potential pharmacological benefit. Finally, we must consider that neurophysiological impairments may be causal to, or as a result of, a myriad of other equally known disease processes that include mitochondrial dysfunction, axonal transport dysregulation, impaired proteostasis and aberrant RNA metabolism. Although future studies have much to elucidate, it is now clear that altered neurophysiological function in *C9ORF72*^RE^ ALS-FTD plays a key role in the pathogenesis of the disease.

## Author Contributions

ISP and ML wrote and edited the manuscript. MSA, CS, and LF edited the final version of the manuscript. All the authors contributed to the article and approved the submitted version.

## Conflict of Interest

The authors declare that the research was conducted in the absence of any commercial or financial relationships that could be construed as a potential conflict of interest.

## Publisher’s Note

All claims expressed in this article are solely those of the authors and do not necessarily represent those of their affiliated organizations, or those of the publisher, the editors and the reviewers. Any product that may be evaluated in this article, or claim that may be made by its manufacturer, is not guaranteed or endorsed by the publisher.

## References

[B1] Agarwal S. Highton-Williamson E. Caga J. Howells J. Dharmadasa T. Matamala J. M. (2021). Motor cortical excitability predicts cognitive phenotypes in amyotrophic lateral sclerosis. *Sci. Rep.* 11 1–9. 10.1038/s41598-021-81612-x 33500476 PMC7838179

[B2] Agosta F. Ferraro P. M. Riva N. Spinelli E. G. Chiò A. Canu E. (2016). Structural brain correlates of cognitive and behavioral impairment in MND. *Hum. Brain Mapp.* 37:1614. 10.1002/HBM.23124 26833930 PMC6867462

[B3] Ahmed T. Van der Jeugd A. Blum D. Galas M. C. D’Hooge R. Buee L. (2014). Cognition and hippocampal synaptic plasticity in mice with a homozygous tau deletion. *Neurobiol. Aging* 35 2474–2478. 10.1016/j.neurobiolaging.2014.05.005 24913895

[B4] Allen S. P. Hall B. Castelli L. M. Francis L. Woof R. Siskos A. P. (2019a). Astrocyte adenosine deaminase loss increases motor neuron toxicity in amyotrophic lateral sclerosis. *Brain* 142 586–605. 10.1093/brain/awy353 30698736 PMC6391613

[B5] Allen S. P. Hall B. Woof R. Francis L. Gatto N. Shaw A. C. (2019b). C9orf72 expansion within astrocytes reduces metabolic flexibility in amyotrophic lateral sclerosis. *Brain* 142 3771–3790. 10.1093/brain/awz302 31647549 PMC6906594

[B6] Allodi I. Montañana-Rosell R. Selvan R. Löw P. Kiehn O. (2021). Locomotor deficits in a mouse model of ALS are paralleled by loss of V1-interneuron connections onto fast motor neurons. *Nat. Commun.* 12 1–18. 10.1038/s41467-021-23224-7 34059686 PMC8166981

[B7] Andersen J. Revah O. Miura Y. Thom N. Amin N. D. Kelley K. W. (2020). Generation of Functional Human 3D Cortico-Motor Assembloids. *Cell* 183 1913.e–1929.e. 10.1016/j.cell.2020.11.017 33333020 PMC8711252

[B8] Aoki Y. Manzano R. Lee Y. Dafinca R. Aoki M. Douglas A. G. L. (2017). C9orf72 and RAB7L1 regulate vesicle trafficking in amyotrophic lateral sclerosis and frontotemporal dementia. *Brain* 140 887–897. 10.1093/brain/awx024 28334866

[B9] Arai T. Hasegawa M. Akiyama H. Ikeda K. Nonaka T. Mori H. (2006). TDP-43 is a component of ubiquitin-positive tau-negative inclusions in frontotemporal lobar degeneration and amyotrophic lateral sclerosis. *Biochem. Biophys. Res. Commun.* 351 602–611. 10.1016/j.bbrc.2006.10.093 17084815

[B10] Bae J. S. Simon N. G. Menon P. Vucic S. Kiernan M. C. (2013). The puzzling case of hyperexcitability in amyotrophic lateral sclerosis. *J. Clin. Neurol.* 9 65–74. 10.3988/jcn.2013.9.2.65 23626643 PMC3633193

[B11] Balendra R. Isaacs A. M. (2018). C9orf72-mediated ALS and FTD: multiple pathways to disease. *Nat. Rev. Neurol.* 14 544–558. 10.1038/s41582-018-0047-2 30120348 PMC6417666

[B12] Barbier M. Camuzat A. Hachimi K. El, Guegan J. Rinaldi D. (2021). SLITRK2, an X-linked modifier of the age at onset in C9orf72 frontotemporal lobar degeneration. *Brain* 144 2798–2811. 10.1093/brain/awab171 34687211

[B13] Benussi A. Cosseddu M. Filareto I. Dell’Era V. Archetti S. Sofia Cotelli M. (2016). Impaired long-term potentiation–like cortical plasticity in presymptomatic genetic frontotemporal dementia. *Ann. Neurol.* 80 472–476. 10.1002/ana.24731 27438089

[B14] Biundo F. Del Prete D. Zhang H. Arancio O. D’Adamio L. (2018). A role for tau in learning, memory and synaptic plasticity. *Sci. Rep.* 8 1–13. 10.1038/s41598-018-21596-3 29453339 PMC5816660

[B15] Burke R. E. (1981). Motor Units: Anatomy, Physiology, and Functional Organization. *Compr. Physiol.* 1981 345–422. 10.1002/cphy.cp010210

[B16] Bursch F. Kalmbach N. Naujock M. Staege S. Eggenschwiler R. Abo-Rady M. (2019). Altered calcium dynamics and glutamate receptor properties in iPSC-derived motor neurons from ALS patients with C9orf72, FUS, SOD1 or TDP43 mutations. *Hum. Mol. Genet.* 28 2835–2850. 10.1093/hmg/ddz107 31108504

[B17] Butti Z. Pan Y. E. Giacomotto J. Patten S. A. (2021). Reduced C9orf72 function leads to defective synaptic vesicle release and neuromuscular dysfunction in zebrafish. *Commun. Biol.* 4:792. 10.1038/S42003-021-02302-Y 34172817 PMC8233344

[B18] Cappello V. Francolini M. (2017). Neuromuscular junction dismantling in amyotrophic lateral sclerosis. *Int. J. Mol. Sci.* 18:ijms18102092. 10.3390/ijms18102092 28972545 PMC5666774

[B19] Castelli L. M. Cutillo L. Souza C. D. S. Sanchez-Martinez A. Granata I. Lin Y. H. (2021). SRSF1-dependent inhibition of C9ORF72-repeat RNA nuclear export: genome-wide mechanisms for neuroprotection in amyotrophic lateral sclerosis. *Mol. Neurodegener.* 16:475–y. 10.1186/s13024-021-00475-y 34376242 PMC8353793

[B20] Catanese A. Rajkumar S. Sommer D. Freisem D. Wirth A. Aly A. (2021). Synaptic disruption and CREB-regulated transcription are restored by K + channel blockers in ALS. *EMBO Mol. Med.* 13:e13131. 10.15252/emmm.202013131 34125498 PMC8261490

[B21] Choi S. Y. Lopez-Gonzalez R. Krishnan G. Phillips H. L. Li A. N. Seeley W. W. (2019). C9ORF72-ALS/FTD-associated poly(GR) binds Atp5a1 and compromises mitochondrial function in vivo. *Nat. Neurosci.* 22 851–862. 10.1038/s41593-019-0397-0 31086314 PMC6800116

[B22] Christidi F. Karavasilis E. Rentzos M. Kelekis N. Evdokimidis I. Bede P. (2018). Clinical and radiological markers of extra-motor deficits in amyotrophic lateral sclerosis. *Front. Neurol.* 9:1005. 10.3389/fneur.2018.01005 30524366 PMC6262087

[B23] Cleveland D. W. Rothstein J. D. (2001). From charcot to lou gehrig: deciphering selective motor neuron death in als. *Nat. Rev. Neurosci.* 2 806–819. 10.1038/35097565 11715057

[B24] Consonni M. Contarino V. E. Catricalà E. Bella E. D. Pensato V. Gellera C. (2018). Cortical markers of cognitive syndromes in amyotrophic lateral sclerosis. *Neuroimage* 19:675. 10.1016/J.NICL.2018.05.020 30023173 PMC6046611

[B25] Couratier P. Sindou P. Hugon J. Couratier P. Hugon J. Vallat J. M. (1993). Cell culture evidence for neuronal degeneration in amyotrophic lateral sclerosis being linked to glutamate AMPA/kainate receptors. *Lancet* 341 265–268. 10.1016/0140-6736(93)92615-Z 8093916

[B26] Coyne A. N. Lorenzini I. Chou C. C. Torvund M. Rogers R. S. Starr A. (2017). Post-transcriptional Inhibition of Hsc70-4/HSPA8 Expression Leads to Synaptic Vesicle Cycling Defects in Multiple Models of ALS. *Cell Rep.* 21 110–125. 10.1016/J.CELREP.2017.09.028 28978466 PMC5679478

[B27] Daube J. R. (1985). Electrophysiologic studies in the diagnosis and prognosis of motor neuron diseases. *Neurol. Clin.* 3 473–493. 10.1016/s0733-8619(18)31017-x 3900681

[B28] de Carvalho M. Swash M. (2016). Lower motor neuron dysfunction in ALS. *Clin. Neurophysiol.* 127 2670–2681. 10.1016/j.clinph.2016.03.024 27117334

[B29] DeJesus-Hernandez M. Mackenzie I. R. Boeve B. F. Boxer A. L. Baker M. Rutherford N. J. (2011). Expanded GGGGCC Hexanucleotide Repeat in Noncoding Region of C9ORF72 Causes Chromosome 9p-Linked FTD and ALS. *Neuron* 72 245–256. 10.1016/j.neuron.2011.09.011 21944778 PMC3202986

[B30] Devlin A. C. Burr K. Borooah S. Foster J. D. Cleary E. M. Geti I. (2015). Human iPSC-derived motoneurons harbouring TARDBP or C9ORF72 ALS mutations are dysfunctional despite maintaining viability. *Nat. Commun.* 6 1–12. 10.1038/ncomms6999 25580746 PMC4338554

[B31] Donnelly C. J. Zhang P. W. Pham J. T. Heusler A. R. Mistry N. A. Vidensky S. (2013). RNA Toxicity from the ALS/FTD C9ORF72 Expansion Is Mitigated by Antisense Intervention. *Neuron* 80 415–428. 10.1016/j.neuron.2013.10.015 24139042 PMC4098943

[B32] Dyer M. S. Woodhouse A. Blizzard C. A. (2021). Cytoplasmic human tdp-43 mislocalization induces widespread dendritic spine loss in mouse upper motor neurons. *Brain Sci.* 11:brainsci11070883. 10.3390/brainsci11070883 34209287 PMC8301870

[B33] Eisen A. Braak H. Tredici K. Del, Lemon R. Ludolph A. C. (2017). Cortical influences drive amyotrophic lateral sclerosis. *J. Neurol. Neurosurg. Psychiatry* 88 917–924. 10.1136/jnnp-2017-315573 28710326

[B34] Estebanez L. Hoffmann D. Voigt B. C. Poulet J. F. A. (2017). Parvalbumin-Expressing GABAergic Neurons in Primary Motor Cortex Signal Reaching. *Cell Rep.* 20 308–318. 10.1016/j.celrep.2017.06.044 28700934 PMC5522533

[B35] Farrant M. Nusser Z. (2005). Variations on an inhibitory theme: Phasic and tonic activation of GABA A receptors. *Nat. Rev. Neurosci.* 6 215–229. 10.1038/nrn1625 15738957

[B36] Fogarty M. J. Klenowski P. M. Lee J. D. Drieberg-Thompson J. R. Bartlett S. E. Ngo S. T. (2016a). Cortical synaptic and dendritic spine abnormalities in a presymptomatic TDP-43 model of amyotrophic lateral sclerosis. *Sci. Rep.* 61 1–13. 10.1038/srep37968 27897242 PMC5126629

[B37] Fogarty M. J. Mu E. W. H. Lavidis N. A. Noakes P. G. Bellingham M. C. (2017). Motor areas show altered dendritic structure in an amyotrophic lateral sclerosis mouse model. *Front. Neurosci.* 11:1–16. 10.3389/fnins.2017.00609 29163013 PMC5672020

[B38] Fogarty M. J. Mu E. W. H. Noakes P. G. Lavidis N. A. Bellingham M. C. (2016b). Marked changes in dendritic structure and spine density precede significant neuronal death in vulnerable cortical pyramidal neuron populations in the SOD1G93A mouse model of amyotrophic lateral sclerosis. *Acta Neuropathol. Commun.* 4:347–y. 10.1186/s40478-016-0347-y 27488828 PMC4973034

[B39] Freibaum B. D. Lu Y. Lopez-Gonzalez R. Kim N. C. Almeida S. Lee K. H. (2015). GGGGCC repeat expansion in C9orf72 compromises nucleocytoplasmic transport. *Nature* 525 129–133. 10.1038/nature14974 26308899 PMC4631399

[B40] Frere S. Slutsky I. (2018). Alzheimer’s Disease: From Firing Instability to Homeostasis Network Collapse. *Neuron* 97 32–58. 10.1016/j.neuron.2017.11.028 29301104

[B41] Frick P. Sellier C. Mackenzie I. R. A. Cheng C. Y. Tahraoui-Bories J. Martinat C. (2018). Novel antibodies reveal presynaptic localization of C9orf72 protein and reduced protein levels in C9orf72 mutation carriers. *Acta Neuropathol. Commun.* 6:72. 10.1186/s40478-018-0579-0 30075745 PMC6091050

[B42] Fritz E. Izaurieta P. Weiss A. Mir F. R. Rojas P. Gonzalez D. (2013). Mutant SOD1-expressing astrocytes release toxic factors that trigger motoneuron death by inducing hyperexcitability. *J. Neurophysiol.* 109 2803–2814. 10.1152/jn.00500.2012 23486205 PMC3680799

[B43] García-Cabrero A. M. Guerrero-López R. Giráldez B. G. Llorens-Martín M. Ávila J. Serratosa J. M. (2013). Hyperexcitability and epileptic seizures in a model of frontotemporal dementia. *Neurobiol. Dis.* 58 200–208. 10.1016/j.nbd.2013.06.005 23774255

[B44] Geevasinga N. Menon P. Howells J. Nicholson G. A. Kiernan M. C. Vucic S. (2015). Axonal ion channel dysfunction in C9orf72 familial amyotrophic lateral sclerosis. *JAMA Neurol.* 72 49–57. 10.1001/jamaneurol.2014.2940 25384182

[B45] Geevasinga N. Menon P. Özdinler P. H. Kiernan M. C. Vucic S. (2016). Pathophysiological and diagnostic implications of cortical dysfunction in ALS. *Nat. Rev. Neurol.* 12 651–661. 10.1038/nrneurol.2016.140 27658852

[B46] Genç B. B. Jara J. H. Lagrimas A. K. B. B. Pytel P. Roos R. P. Mesulam M. M. (2017). Apical dendrite degeneration, a novel cellular pathology for Betz cells in ALS. *Sci. Rep.* 7 1–10. 10.1038/srep41765 28165465 PMC5292972

[B47] Gendron T. F. Belzil V. V. Zhang Y. J. Petrucelli L. (2014). Mechanisms of toxicity in C9FTLD/ALS. *Acta Neuropathol.* 127 359–376. 10.1007/s00401-013-1237-z 24394885 PMC4002260

[B48] Gendron T. F. Bieniek K. F. Zhang Y.-J. Jansen-West K. Ash P. E. A. Caulfield T. (2013). Antisense transcripts of the expanded C9ORF72 hexanucleotide repeat form nuclear RNA foci and undergo repeat-associated non-ATG translation in c9FTD/ALS. *Acta Neuropathol.* 126 829–844. 10.1007/s00401-013-1192-8 24129584 PMC3830741

[B49] Gerges N. Z. Backos D. S. Esteban J. A. (2004). Local control of AMPA receptor trafficking at the postsynaptic terminal by a small GTPase of the Rab family. *J. Biol. Chem.* 279 43870–43878. 10.1074/jbc.M404982200 15297461

[B50] Ghatak S. Dolatabadi N. Trudler D. Zhang X. Wu Y. Mohata M. (2019). Mechanisms of hyperexcitability in alzheimer’s disease hiPSC-derived neurons and cerebral organoids vs. Isogenic control. *Elife* 8:50333. 10.7554/ELIFE.50333 31782729 PMC6905854

[B51] Gorrie G. H. Fecto F. Radzicki D. Weiss C. Shi Y. Dong H. (2014). Dendritic spinopathy in transgenic mice expressing ALS/dementia-linked mutant UBQLN2. *Proc. Natl. Acad. Sci. U S A.* 111 14524–14529. 10.1073/pnas.1405741111 25246588 PMC4209984

[B52] Gregory J. M. Livesey M. R. McDade K. Selvaraj B. T. Barton S. K. Chandran S. (2020). Dysregulation of AMPA receptor subunit expression in sporadic ALS post-mortem brain. *J. Pathol.* 250 67–78. 10.1002/path.5351 31579943 PMC6973025

[B53] Gunes Z. I. Kan V. W. Y. Ye X. Q. Liebscher S. (2020). Exciting Complexity: The Role of Motor Circuit Elements in ALS Pathophysiology. *Front. Neurosci.* 14:573. 10.3389/fnins.2020.00573 32625051 PMC7311855

[B54] Guo W. Naujock M. Fumagalli L. Vandoorne T. Baatsen P. Boon R. (2017). HDAC6 inhibition reverses axonal transport defects in motor neurons derived from FUS-ALS patients. *Nat. Commun.* 8:911–y. 10.1038/s41467-017-00911-y 29021520 PMC5636840

[B55] Haidar M. Viden A. Cuic B. Wang T. Rosier M. Tomas D. (2021). Cortical hyperexcitability drives dying forward ALS symptoms and pathology in mice. *bioRxiv* [Preprint]. 10.1101/2021.08.13.456320 40784541

[B56] Hammer R. P. Tomiyasu U. Scheibel A. B. (1979). Degeneration of the human Betz cell due to amyotrophic lateral sclerosis. *Exp. Neurol.* 63 336–346. 10.1016/0014-4886(79)90129-8 437007

[B57] Handley E. E. Pitman K. A. Dawkins E. Young K. M. Clark R. M. Jiang T. C. (2017). Synapse Dysfunction of Layer v Pyramidal Neurons Precedes Neurodegeneration in a Mouse Model of TDP-43 Proteinopathies. *Cereb. Cortex* 27 3630–3647. 10.1093/cercor/bhw185 27496536

[B58] Henstridge C. M. Sideris D. I. Carroll E. Rotariu S. Salomonsson S. Tzioras M. (2018). Synapse loss in the prefrontal cortex is associated with cognitive decline in amyotrophic lateral sclerosis. *Acta Neuropathol.* 135 213–226. 10.1007/s00401-017-1797-4 29273900 PMC5773656

[B59] Henstridge C. M. Tzioras M. Paolicelli R. C. (2019). Glial contribution to excitatory and inhibitory synapse loss in neurodegeneration. *Front. Cell. Neurosci.* 13:63. 10.3389/fncel.2019.00063 30863284 PMC6399113

[B60] Ho W. Y. Navakkode S. Liu F. Soong T. W. Ling S. C. (2020). Deregulated expression of a longevity gene, Klotho, in the C9orf72 deletion mice with impaired synaptic plasticity and adult hippocampal neurogenesis. *Acta Neuropathol. Commun.* 8:155. 10.1186/s40478-020-01030-4 32887666 PMC7473815

[B61] Hossaini M. Cano S. C. Van Dis V. Haasdijk E. D. Hoogenraad C. C. Holstege J. C. (2011). Spinal inhibitory interneuron pathology follows motor neuron degeneration independent of glial mutant superoxide dismutase 1 expression in SOD1-ALS mice. *J. Neuropathol. Exp. Neurol.* 70 662–677. 10.1097/NEN.0b013e31822581ac 21760539

[B62] Huang X. Roet K. C. D. Zhang L. Brault A. Berg A. P. Jefferson A. B. (2021). Human amyotrophic lateral sclerosis excitability phenotype screen: Target discovery and validation. *Cell Rep.* 35:109224. 10.1016/j.celrep.2021.109224 34107252 PMC8209673

[B63] Iwai Y. Shibuya K. Misawa S. Sekiguchi Y. Watanabe K. Amino H. (2016). Axonal dysfunction precedes motor neuronal death in amyotrophic lateral sclerosis. *PLoS One* 11:0158596. 10.1371/journal.pone.0158596 27383069 PMC4934877

[B64] Jensen B. K. Schuldi M. H. McAvoy K. Russell K. A. Boehringer A. Curran B. M. (2020). Synaptic dysfunction induced by glycine-alanine dipeptides in C9orf72- ALS / FTD is rescued by SV 2 replenishment. *EMBO Mol. Med.* 12:201910722. 10.15252/emmm.201910722 32347002 PMC7207170

[B65] Kato S. Hayashi H. Yagishita A. (1993). Involvement of the frontotemporal lobe and limbic system in amyotrophic lateral sclerosis: As assessed by serial computed tomography and magnetic resonance imaging. *J. Neurol. Sci.* 116 52–58. 10.1016/0022-510X(93)90089-H 8509805

[B66] Kawahara Y. Ito K. Sun H. Aizawa H. Kanazawa I. Kwak S. (2004a). RNA editing and death of motor neurons: There is a glutamate-receptor defect in patients with amyotrophic lateral sclerosis. *Nature* 427:801. 10.1038/427801a 14985749

[B67] Kawahara Y. Ito K. Sun H. Ito M. Kanazawa I. Kwak S. (2004b). Regulation of glutamate receptor RNA editing and ADAR mRNA expression in developing human normal and Down’s syndrome brains. *Dev. Brain Res.* 148 151–155. 10.1016/j.devbrainres.2003.11.008 14757529

[B68] Kelley K. W. Ben Haim L. Schirmer L. Tyzack G. E. Tolman M. Miller J. G. (2018). Kir4.1-Dependent Astrocyte-Fast Motor Neuron Interactions Are Required for Peak Strength. *Neuron* 98 306.e–319.e. 10.1016/j.neuron.2018.03.010 29606582 PMC5919779

[B69] Kim J. Hughes E. G. Shetty A. S. Arlotta P. Goff L. A. Bergles D. E. (2017). Changes in the excitability of neocortical neurons in a mouse model of amyotrophic lateral sclerosis are not specific to corticospinal neurons and are modulated by advancing disease. *J. Neurosci.* 37 9037–9053. 10.1523/JNEUROSCI.0811-17.2017 28821643 PMC5597984

[B70] Koza P. Beroun A. Konopka A. Górkiewicz T. Bijoch L. Torres J. C. (2019). Neuronal TDP-43 depletion affects activity-dependent plasticity. *Neurobiol. Dis.* 130:104499. 10.1016/j.nbd.2019.104499 31176717

[B71] Kujirai T. Caramia M. D. Rothwell J. C. Day B. L. Thompson P. D. Ferbert A. (1993). Corticocortical inhibition in human motor cortex. *J. Physiol.* 471 501–519. 10.1113/jphysiol.1993.sp019912 8120818 PMC1143973

[B72] Lall D. Lorenzini I. Mota T. A. Bell S. Mahan T. E. Ulrich J. D. (2021). C9orf72 deficiency promotes microglial-mediated synaptic loss in aging and amyloid accumulation. *Neuron* 109 2275.e–2291.e. 10.1016/j.neuron.2021.05.020 34133945 PMC8298293

[B73] Lemon R. N. (2008). Descending pathways in motor control. *Annu. Rev. Neurosci.* 31 195–218. 10.1146/annurev.neuro.31.060407.125547 18558853

[B74] Leroy F. Zytnicki D. (2015). Is hyperexcitability really guilty in amyotrophic lateral sclerosis? *Neural Regen. Res.* 10 1413–1415. 10.4103/1673-5374.165308 26604899 PMC4625504

[B75] Lin Z. Kim E. Ahmed M. Han G. Simmons C. Redhead Y. (2021). MRI-guided histology of TDP-43 knock-in mice implicates parvalbumin interneuron loss, impaired neurogenesis and aberrant neurodevelopment in amyotrophic lateral sclerosis-frontotemporal dementia. *Brain Commun.* 3:fcab114. 10.1093/braincomms/fcab114 34136812 PMC8204366

[B76] Lindau M. Jelic V. Johansson S. E. Andersen C. Wahlund L. O. Almkvist O. (2003). Quantitative EEG abnormalities and cognitive dysfunctions in frontotemporal dementia and Alzheimer’s disease. *Dement. Geriatr. Cogn. Disord.* 15 106–114. 10.1159/000067973 12566600

[B77] Livesey M. R. Magnani D. Hardingham G. E. Chandran S. Wyllie D. J. A. (2016). Functional properties of in vitro excitatory cortical neurons derived from human pluripotent stem cells. *J. Physiol.* 594 6573–6582. 10.1113/JP270660 26608229 PMC5108911

[B78] Lomen-Hoerth C. Anderson T. Miller B. (2002). The overlap of amyotrophic lateral sclerosis and frontotemporal dementia. *Neurology* 59 1077–1079. 10.1212/WNL.59.7.1077 12370467

[B79] Lui H. Zhang J. Makinson S. R. Cahill M. K. Kelley K. W. Huang H. Y. (2016). Progranulin Deficiency Promotes Circuit-Specific Synaptic Pruning by Microglia via Complement Activation. *Cell* 165 921–935. 10.1016/j.cell.2016.04.001 27114033 PMC4860138

[B80] Malenka R. C. Bear M. F. (2004). LTP and LTD: An Embarrassment of Riches. *Neuron* 44 5–21. 10.1016/J.NEURON.2004.09.012 15450156

[B81] Martin L. J. Chang Q. (2012). Inhibitory synaptic regulation of motoneurons: A new target of disease mechanisms in amyotrophic lateral sclerosis. *Mol. Neurobiol.* 45 30–42. 10.1007/s12035-011-8217-x 22072396 PMC3530198

[B82] Martínez-Silva M. de L. Imhoff-Manuel R. D. Sharma A. Heckman C. J. Shneider N. A. (2018). Hypoexcitability precedes denervation in the large fast-contracting motor units in two unrelated mouse models of ALS. *Elife* 7:30955. 10.7554/eLife.30955 29580378 PMC5922970

[B83] Maselli R. A. Wollman R. L. Leung C. Distad B. Palombi S. Richman D. P. (1993). Neuromuscular transmission in amyotrophic lateral sclerosis. *Muscle Nerve* 16 1193–1203. 10.1002/mus.880161109 8105377

[B84] McColgan P. Joubert J. Tabrizi S. J. Rees G. (2020). The human motor cortex microcircuit: insights for neurodegenerative disease. *Nat. Rev. Neurosci.* 21 401–415. 10.1038/s41583-020-0315-1 32555340

[B85] Menon P. Geevasinga N. van den Bos M. Yiannikas C. Kiernan M. C. Vucic S. (2017). Cortical hyperexcitability and disease spread in amyotrophic lateral sclerosis. *Eur. J. Neurol.* 24 816–824. 10.1111/ene.13295 28436181

[B86] Menon P. Higashihara M. Bos M. van den, Geevasinga N. Kiernan M. C. (2020). Cortical hyperexcitability evolves with disease progression in ALS. *Ann. Clin. Transl. Neurol.* 7:733. 10.1002/ACN3.51039 32304186 PMC7261748

[B87] Menon P. Kiernan M. C. Vucic S. (2015). Cortical hyperexcitability precedes lower motor neuron dysfunction in ALS. *Clin. Neurophysiol.* 126 803–809. 10.1016/j.clinph.2014.04.023 25227219

[B88] Meyer K. Ferraiuolo L. Miranda C. J. Likhite S. McElroy S. Renusch S. (2014). Direct conversion of patient fibroblasts demonstrates non-cell autonomous toxicity of astrocytes to motor neurons in familial and sporadic ALS. *Proc. Natl. Acad. Sci. U S A.* 111 829–832. 10.1073/pnas.1314085111 24379375 PMC3896192

[B89] Mignogna M. L. Giannandrea M. Gurgone A. Fanelli F. Raimondi F. Mapelli L. (2015). The intellectual disability protein RAB39B selectively regulates GluA2 trafficking to determine synaptic AMPAR composition. *Nat. Commun.* 6 1–15. 10.1038/ncomms7504 25784538 PMC4383008

[B90] Mignogna M. L. Musardo S. Ranieri G. Gelmini S. Espinosa P. Marra P. (2021). RAB39B-mediated trafficking of the GluA2-AMPAR subunit controls dendritic spine maturation and intellectual disability-related behaviour. *Mol. Psychiatry* 2021 1–19. 10.1038/s41380-021-01155-5 34035473 PMC8760075

[B91] Milnerwood A. J. Raymond L. A. (2010). Early synaptic pathophysiology in neurodegeneration: Insights from Huntington’s disease. *Trends Neurosci.* 33 513–523. 10.1016/j.tins.2010.08.002 20850189

[B92] Mizielinska S. Isaacs A. M. (2014). C9orf72 amyotrophic lateral sclerosis and frontotemporal dementia: Gain or loss of function? *Curr. Opin. Neurol.* 27 515–523. 10.1097/WCO.0000000000000130 25188012 PMC4165481

[B93] Mogyoros I. Kiernan M. C. Burke D. Bostock H. (1998). Strength-duration properties of sensory and motor axons in amyotrophic lateral sclerosis. *Brain* 121 851–859. 10.1093/brain/121.5.851 9619189

[B94] Mohammadi B. Kollewe K. Cole D. M. Fellbrich A. Heldmann M. Samii A. (2015). Amyotrophic lateral sclerosis affects cortical and subcortical activity underlying motor inhibition and action monitoring. *Hum. Brain Mapp.* 36 2878–2889. 10.1002/hbm.22814 25913637 PMC6869134

[B95] Moore S. Alsop E. Lorenzini I. Starr A. Rabichow B. E. Mendez E. (2019). ADAR2 mislocalization and widespread RNA editing aberrations in C9orf72-mediated ALS/FTD. *Acta Neuropathol.* 138:1999–w. 10.1007/s00401-019-01999-w 30945056 PMC6750285

[B96] Mori K. Weng S. M. Arzberger T. May S. Rentzsch K. Kremmer E. (2013). The C9orf72 GGGGCC repeat is translated into aggregating dipeptide-repeat proteins in FTLD/ALS. *Science* 339 1335–1338. 10.1126/science.1232927 23393093

[B97] Nasseroleslami B. Dukic S. Broderick M. Mohr K. Schuster C. Gavin B. (2019). Characteristic Increases in EEG Connectivity Correlate with Changes of Structural MRI in Amyotrophic Lateral Sclerosis. *Cereb. Cortex* 29 27–41. 10.1093/cercor/bhx301 29136131

[B98] Naujock M. Stanslowsky N. Bufler S. Naumann M. Reinhardt P. Sterneckert J. (2016). 4-Aminopyridine Induced Activity Rescues Hypoexcitable Motor Neurons from Amyotrophic Lateral Sclerosis Patient-Derived Induced Pluripotent. *Stem Cells* 34 1563–1575. 10.1002/stem.2354 26946488

[B99] Neumann M. Sampathu D. M. Kwong L. K. Truax A. C. Micsenyi M. C. Chou T. T. (2006). Ubiquitinated TDP-43 in frontotemporal lobar degeneration and amyotrophic lateral sclerosis. *Science* 314 130–133. 10.1126/science.1134108 17023659

[B100] Nishida K. Yoshimura M. Isotani T. Yoshida T. Kitaura Y. Saito A. (2011). Differences in quantitative EEG between frontotemporal dementia and Alzheimer’s disease as revealed by LORETA. *Clin. Neurophysiol.* 122 1718–1725. 10.1016/j.clinph.2011.02.011 21396882

[B101] Niu M. Zheng N. Wang Z. Gao Y. Luo X. Chen Z. (2020). RAB39B Deficiency Impairs Learning and Memory Partially Through Compromising Autophagy. *Front. Cell Dev. Biol.* 8:1508. 10.3389/fcell.2020.598622 33364235 PMC7753041

[B102] Palop J. J. Chin J. Mucke L. (2006). A network dysfunction perspective on neurodegenerative diseases. *Nature* 443 768–773. 10.1038/nature05289 17051202

[B103] Perkins E. M. Burr K. Banerjee P. Mehta A. R. Dando O. Selvaraj B. T. (2021). Altered network properties in C9ORF72 repeat expansion cortical neurons are due to synaptic dysfunction. *Mol. Neurodegener.* 16:13. 10.1186/s13024-021-00433-8 33663561 PMC7931347

[B104] Perry S. Han Y. Das A. Dickman D. (2017). Homeostatic plasticity can be induced and expressed to restore synaptic strength at neuromuscular junctions undergoing ALS-related degeneration. *Hum. Mol. Genet.* 26 4153–4167. 10.1093/hmg/ddx304 28973139 PMC5886083

[B105] Peters O. M. Cabrera G. T. Tran H. Gendron T. F. McKeon J. E. Metterville J. (2015). Human C9ORF72 Hexanucleotide Expansion Reproduces RNA Foci and Dipeptide Repeat Proteins but Not Neurodegeneration in BAC Transgenic Mice. *Neuron* 88 902–909. 10.1016/j.neuron.2015.11.018 26637797 PMC4828340

[B106] Petkau T. L. Neal S. J. Milnerwood A. Mew A. Hill A. M. Orban P. (2012). Synaptic dysfunction in progranulin-deficient mice. *Neurobiol. Dis.* 45 711–722. 10.1016/J.NBD.2011.10.016 22062772

[B107] Pina-Crespo J. C. Sanz-Blasco S. Lipton S. A. (2014). Concept of excitotoxicity via glutamate receptors. *Handb. Neurotox.* 2 1015–1038. 10.1007/978-1-4614-5836-4_125

[B108] Proudfoot M. Rohenkohl G. Quinn A. Colclough G. L. Wuu J. Talbot K. (2016). Altered cortical beta-band oscillations reflect motor system degeneration in amyotrophic lateral sclerosis. *Hum. Brain Mapp.* 38 237–254. 10.1002/hbm.23357 27623516 PMC5215611

[B109] Prudencio M. Belzil V. V. Batra R. Ross C. A. Gendron T. F. Pregent L. J. (2015). Distinct brain transcriptome profiles in C9orf72-associated and sporadic ALS. *Nat. Neurosci.* 18:1175. 10.1038/NN.4065 26192745 PMC4830686

[B110] Renton A. E. Majounie E. Waite A. Simón-Sánchez J. Rollinson S. Gibbs J. R. (2011). A hexanucleotide repeat expansion in C9ORF72 is the cause of chromosome 9p21-linked ALS-FTD. *Neuron* 72 257–268. 10.1016/j.neuron.2011.09.010 21944779 PMC3200438

[B111] Rizzu P. Blauwendraat C. Heetveld S. Lynes E. M. Castillo-Lizardo M. Dhingra A. (2016). C9orf72 is differentially expressed in the central nervous system and myeloid cells and consistently reduced in C9orf72, MAPT and GRN mutation carriers. *Acta Neuropathol. Commun.* 4 37. 10.1186/s40478-016-0306-7 27079381 PMC4832459

[B112] Rosenblum L. T. Trotti D. (2017). EAAT2 and the molecular signature of amyotrophic lateral sclerosis. *Adv. Neurobiol.* 16 117–136. 10.1007/978-3-319-55769-4_6 28828608 PMC6668619

[B113] Rothstein J. D. (1995). Excitotoxic mechanisms in the pathogenesis of amyotrophic lateral sclerosis. *Adv. Neurol.* 68 7–20. 8787245

[B114] Rothstein J. D. Martin L. J. Kuncl R. W. (1992). Decreased Glutamate Transport by the Brain and Spinal Cord in Amyotrophic Lateral Sclerosis. *N. Engl. J. Med.* 326 1464–1468. 10.1056/nejm199205283262204 1349424

[B115] Rothstein J. D. Tsai G. Kuncl R. W. Clawson L. Cornblath D. R. Drachman D. B. (1990). Abnormal excitatory amino acid metabolism in amyotrophic lateral sclerosis. *Ann. Neurol.* 28 18–25. 10.1002/ana.410280106 2375630

[B116] Saba L. Viscomi M. T. Caioli S. Pignataro A. Bisicchia E. Pieri M. (2016). Altered Functionality, Morphology, and Vesicular Glutamate Transporter Expression of Cortical Motor Neurons from a Presymptomatic Mouse Model of Amyotrophic Lateral Sclerosis. *Cereb. Cortex* 26 1512–1528. 10.1093/cercor/bhu317 25596588

[B117] Sahadevan S. Hembach K. M. Tantardini E. Pérez-Berlanga M. Hruska-Plochan M. Megat S. (2021). Synaptic FUS accumulation triggers early misregulation of synaptic RNAs in a mouse model of ALS. *Nat. Commun.* 12 1–17. 10.1038/s41467-021-23188-8 34021139 PMC8140117

[B118] Sahara Khademullah C. Aqrabawi A. J. Place K. M. Dargaei Z. Liang X. Pressey J. C. (2020). Cortical interneuron-mediated inhibition delays the onset of amyotrophic lateral sclerosis. *Brain* 143 800–810. 10.1093/brain/awaa034 32203578

[B119] Sances S. Bruijn L. I. Chandran S. Eggan K. Ho R. Klim J. R. (2016). Modeling ALS with motor neurons derived from human induced pluripotent stem cells. *Nat. Neurosci.* 19 542–553. 10.1038/nn.4273 27021939 PMC5015775

[B120] Sareen D. O’Rourke J. G. Meera P. Muhammad A. K. M. G. Grant S. Simpkinson M. (2013). Targeting RNA foci in iPSC-derived motor neurons from ALS patients with a C9ORF72 repeat expansion. *Sci. Transl. Med.* 5:208ra149. 10.1126/scitranslmed.3007529 24154603 PMC4090945

[B121] Sasaki S. Maruyama S. (1994). Immunocytochemical and ultrastructural studies of the motor cortex in amyotrophic lateral sclerosis. *Acta Neuropathol.* 87 578–585. 10.1007/BF00293318 8091950

[B122] Scekic-Zahirovic J. Sanjuan-Ruiz I. Kan V. Megat S. De Rossi P. Dieterlé S. (2021). Cytoplasmic FUS triggers early behavioral alterations linked to cortical neuronal hyperactivity and inhibitory synaptic defects. *Nat. Commun.* 12 1–19. 10.1038/s41467-021-23187-9 34021132 PMC8140148

[B123] Schanz O. Bageac D. Braun L. Traynor B. J. Lehky T. J. Floeter M. K. (2016). Cortical hyperexcitability in patients with C9ORF72 mutations: Relationship to phenotype. *Muscle Nerve* 54 264–269. 10.1002/mus.25047 26799151 PMC4940214

[B124] Selkoe D. J. (2002). Alzheimer’s disease is a synaptic failure. *Science* 298 789–791. 10.1126/science.1074069 12399581

[B125] Selvaraj B. T. Livesey M. R. Zhao C. Gregory J. M. James O. T. Cleary E. M. (2018). C9ORF72 repeat expansion causes vulnerability of motor neurons to Ca2+-permeable AMPA receptor-mediated excitotoxicity. *Nat. Commun.* 9 1–14. 10.1038/s41467-017-02729-0 29367641 PMC5783946

[B126] Sephtona C. F. Tangc A. A. Kulkarnia A. Westa J. Brooksa M. Stubblefielda J. J. (2014). Activity-dependent FUS dysregulation disrupts. *Proc. Natl. Acad. Sci. U S A.* 111 E4769–E4778. 10.1073/pnas.1406162111 25324524 PMC4226112

[B127] Serio A. Bilican B. Barmada S. J. Ando D. M. Zhao C. Siller R. (2013). Astrocyte pathology and the absence of non-cell autonomy in an induced pluripotent stem cell model of TDP-43 proteinopathy. *Proc. Natl. Acad. Sci. U S A.* 110 4697–4702. 10.1073/pnas.1300398110 23401527 PMC3607024

[B128] Shao Q. Liang C. Chang Q. Zhang W. Yang M. Chen J. F. (2019). C9orf72 deficiency promotes motor deficits of a C9ALS/FTD mouse model in a dose-dependent manner. *Acta Neuropathol. Commun.* 7:32. 10.1186/s40478-019-0685-7 30832726 PMC6398253

[B129] Shaw P. J. (2005). Molecular and cellular pathways of neurodegeneration in motor neurone disease. *J. Neurol. Neurosurg. Psychiatry* 76 1046–1057. 10.1136/jnnp.2004.048652 16024877 PMC1739758

[B130] Shi Y. Hung S. T. Rocha G. Lin S. Linares G. R. Staats K. A. (2019). Identification and therapeutic rescue of autophagosome and glutamate receptor defects in C9ORF72 and sporadic ALS neurons. *JCI Insight* 4:127736. 10.1172/jci.insight.127736 31310593 PMC6693831

[B131] Shi Y. Lin S. Staats K. A. Li Y. Chang W. H. Hung S. T. (2018). Haploinsufficiency leads to neurodegeneration in C9ORF72 ALS/FTD human induced motor neurons. *Nat. Med.* 24 313–325. 10.1038/nm.4490 29400714 PMC6112156

[B132] Shibuya K. Park S. B. Geevasinga N. Menon P. Howells J. Simon N. G. (2016). Motor cortical function determines prognosis in sporadic ALS. *Neurology* 87 513–520. 10.1212/WNL.0000000000002912 27402895

[B133] Šišková Z. Justus D. Kaneko H. Friedrichs D. Henneberg N. Beutel T. (2014). Dendritic structural degeneration is functionally linked to cellular hyperexcitability in a mouse model of alzheimer’s disease. *Neuron* 84 1023–1033. 10.1016/j.neuron.2014.10.024 25456500

[B134] Smeyers J. Banchi E. G. Latouche M. (2021). C9ORF72: What It Is, What It Does, and Why It Matters. *Front. Cell Neurosci.* 15:109. 10.3389/fncel.2021.661447 34025358 PMC8131521

[B135] Snowden J. S. Rollinson S. Thompson J. C. Harris J. M. Stopford C. L. Richardson A. M. T. (2012). Distinct clinical and pathological characteristics of frontotemporal dementia associated with C9ORF72 mutations. *Brain* 135 693–708. 10.1093/brain/awr355 22300873 PMC3286329

[B136] Spalloni A. Origlia N. Sgobio C. Trabalza A. Nutini M. Berretta N. (2011). Postsynaptic alteration of NR2A subunit and defective autophosphorylation of alpha CaMKII at Threonine-286 contribute to abnormal plasticity and morphology of upper motor neurons in presymptomatic SOD1 G93A mice, a murine model for amyotrophic lateral scl. *Cereb. Cortex* 21 796–805. 10.1093/cercor/bhq152 20732897

[B137] Staats K. A. Seah C. Sahimi A. Wang Y. Koutsodendris N. Lin S. (2019). Small molecule inhibition of PIKFYVE kinase rescues gain- and loss-of-function C9ORF72 ALS/FTD disease processes in vivo. *bioRxiv* 2019:685800. 10.1101/685800

[B138] Starr A. Sattler R. (2018). Synaptic dysfunction and altered excitability in C9ORF72 ALS/FTD. *Brain Res.* 1693 98–108. 10.1016/j.brainres.2018.02.011 29453960 PMC5997509

[B139] Styr B. Slutsky I. (2018). Imbalance between firing homeostasis and synaptic plasticity drives early-phase Alzheimer’s disease. *Nat. Neurosci.* 21 463–473. 10.1038/s41593-018-0080-x 29403035 PMC6533171

[B140] Suminaite D. Lyons D. A. Livesey M. R. (2019). Myelinated axon physiology and regulation of neural circuit function. *Glia* 67 2050–2062. 10.1002/glia.23665 31233642 PMC6772175

[B141] Talbot P. R. Goulding P. J. Lloyd J. J. Snowden J. S. Neary D. Testa H. J. (1995). Inter-relation between “classic” motor neuron disease and frontotemporal dementia: Neuropsychological and single photon emission computed tomography study. *J. Neurol. Neurosurg. Psychiatry* 58 541–547. 10.1136/jnnp.58.5.541 7745399 PMC1073482

[B142] Traynelis S. F. Wollmuth L. P. McBain C. J. Menniti F. S. Vance K. M. Ogden K. K. (2010). Glutamate receptor ion channels: Structure, regulation, and function. *Pharmacol. Rev.* 62 405–496. 10.1124/pr.109.002451 20716669 PMC2964903

[B143] Tsuiji H. Inoue I. Takeuchi M. Furuya A. Yamakage Y. Watanabe S. (2017). TDP-43 accelerates age-dependent degeneration of interneurons. *Sci. Rep.* 7:14966–w. 10.1038/s41598-017-14966-w 29097807 PMC5668320

[B144] Turrigiano G. (2012). Homeostatic synaptic plasticity: Local and global mechanisms for stabilizing neuronal function. *Cold Spring Harb. Perspect. Biol.* 4:a005736. 10.1101/cshperspect.a005736 22086977 PMC3249629

[B145] Udagawa T. Fujioka Y. Tanaka M. Honda D. Yokoi S. Riku Y. (2015). FUS regulates AMPA receptor function and FTLD/ALS-associated behaviour via GluA1 mRNA stabilization. *Nat. Commun.* 6 1–13. 10.1038/ncomms8098 25968143 PMC4479014

[B146] Umpierre A. D. Wu L. J. (2021). How microglia sense and regulate neuronal activity. *Glia* 69 1637–1653. 10.1002/glia.23961 33369790 PMC8113084

[B147] Van Den Bosch L. Van Damme P. Bogaert E. Robberecht W. (2006). The role of excitotoxicity in the pathogenesis of amyotrophic lateral sclerosis. *Biochim. Biophys. Acta Mol. Basis Dis.* 1762 1068–1082. 10.1016/j.bbadis.2006.05.002 16806844

[B148] Van Zundert B. Peuscher M. H. Hynynen M. Chen A. Neve R. L. Brown R. H. (2008). Neonatal neuronal circuitry shows hyperexcitable disturbance in a mouse model of the adult-onset neurodegenerative disease amyotrophic lateral sclerosis. *J. Neurosci.* 28 10864–10874. 10.1523/JNEUROSCI.1340-08.2008 18945894 PMC3844745

[B149] Vucic S. Ziemann U. Eisen A. Hallett M. Kiernan M. C. (2013). Transcranial magnetic stimulation and amyotrophic lateral sclerosis: pathophysiological insights. *J. Neurol. Neurosurg. Psychiatry* 84 1161–1170. 10.1136/JNNP-2012-304019 23264687 PMC3786661

[B150] Wainger B. J. Cudkowicz M. E. (2015). Cortical hyperexcitability in amyotrophic lateral sclerosis C9ORF72 repeats. *JAMA Neurol.* 72 1235–1236. 10.1001/jamaneurol.2015.2197 26348624

[B151] Wainger B. J. Kiskinis E. Mellin C. Wiskow O. Steve S. W. Berry J. D. (2014). Intrinsic membrane hyperexcitability of ALS patient-derived motor neurons. *Cell Rep.* 7 1–11. 10.1016/j.celrep.2014.03.019.Intrinsic 24703839 PMC4023477

[B152] Wainger B. J. Macklin E. A. Vucic S. McIlduff C. E. Paganoni S. Maragakis N. J. (2021). Effect of Ezogabine on Cortical and Spinal Motor Neuron Excitability in Amyotrophic Lateral Sclerosis: A Randomized Clinical Trial. *JAMA Neurol.* 78 186–196. 10.1001/jamaneurol.2020.4300 33226425 PMC7684515

[B153] Weskamp K. Tank E. M. Miguez R. McBride J. P. Gómez N. B. White M. (2020). Shortened TDP43 isoforms upregulated by neuronal hyperactivity drive TDP43 pathology in ALS. *J. Clin. Invest.* 130 1139–1155. 10.1172/JCI130988 31714900 PMC7269575

[B154] West R. J. H. Sharpe J. L. Voelzmann A. Munro A. L. Hahn I. Baines R. A. (2020). Co-expression of C9orf72 related dipeptide-repeats over 1000 repeat units reveals age-A nd combination-specific phenotypic profiles in Drosophila. *Acta Neuropathol. Commun.* 8 1–19. 10.1186/s40478-020-01028-y 32894207 PMC7487709

[B155] Westergard T. McAvoy K. Russell K. Wen X. Pang Y. Morris B. (2019). Repeat-associated non- AUG translation in C9orf72- ALS / FTD is driven by neuronal excitation and stress. *EMBO Mol. Med.* 11:201809423. 10.15252/emmm.201809423 30617154 PMC6365928

[B156] Williams K. L. Fifita J. A. Vucic S. Durnall J. C. Kiernan M. C. Blair I. P. (2013). Pathophysiological insights into ALS with C9ORF72 expansions. *J. Neurol. Neurosurg. Psychiatry* 84 931–935. 10.1136/jnnp-2012-304529 23463871

[B157] Wu L. S. Cheng W. C. Chen C. Y. Wu M. C. Wang Y. C. Tseng Y. H. (2019). Transcriptomopathies of pre- and post-symptomatic frontotemporal dementia-like mice with TDP-43 depletion in forebrain neurons. *Acta Neuropathol. Commun.* 7:50. 10.1186/s40478-019-0674-x 30922385 PMC6440020

[B158] Xiao S. McKeever P. M. Lau A. Robertson J. (2019). Synaptic localization of C9orf72 regulates post-synaptic glutamate receptor 1 levels. *Acta Neuropathol. Commun.* 7:161. 10.1186/s40478-019-0812-5 31651360 PMC6813971

[B159] Xu W. Xu J. (2018). C9orf72 dipeptide repeats cause selective neurodegeneration and cell-autonomous excitotoxicity in Drosophila glutamatergic neurons. *J. Neurosci.* 38 7741–7752. 10.1523/JNEUROSCI.0908-18.2018 30037833 PMC6705968

[B160] Zhang K. Donnelly C. J. Haeusler A. R. Grima J. C. Machamer J. B. Steinwald P. (2015). The C9orf72 repeat expansion disrupts nucleocytoplasmic transport. *Nature* 525 56–61. 10.1038/nature14973 26308891 PMC4800742

[B161] Zhang W. Zhang L. Liang B. Schroeder D. Zhang Z. W. Cox G. A. (2016). Hyperactive somatostatin interneurons contribute to excitotoxicity in neurodegenerative disorders. *Nat. Neurosci.* 19 557–559. 10.1038/nn.4257 26900927 PMC4811704

[B162] Zhang Z. Almeida S. Lu Y. Nishimura A. L. Peng L. Sun D. (2013). Downregulation of MicroRNA-9 in iPSC-Derived Neurons of FTD/ALS Patients with TDP-43 Mutations. *PLoS One* 8:0076055. 10.1371/journal.pone.0076055 24143176 PMC3797144

[B163] Zhao C. Devlin A. C. Chouhan A. K. Selvaraj B. T. Stavrou M. Burr K. (2020). Mutant C9orf72 human iPSC-derived astrocytes cause non-cell autonomous motor neuron pathophysiology. *Glia* 68 1046–1064. 10.1002/glia.23761 31841614 PMC7078830

[B164] Zhu Q. Jiang J. Gendron T. F. McAlonis-Downes M. Jiang L. Taylor A. (2020). Reduced C9ORF72 function exacerbates gain of toxicity from ALS/FTD-causing repeat expansion in C9orf72. *Nat. Neurosci.* 23 615–624. 10.1038/s41593-020-0619-5 32284607 PMC7384305

